# Time Evolution of Bacterial Resistance Observed with Principal Component Analysis

**DOI:** 10.3390/antibiotics14070729

**Published:** 2025-07-20

**Authors:** Claudia P. Barrera Patiño, Mitchell Bonner, Andrew Ramos Borsatto, Jennifer M. Soares, Kate C. Blanco, Vanderlei S. Bagnato

**Affiliations:** 1 Sao Carlos Institute of Physics (IFSC), University of Sao Paulo (USP), Sao Carlos 13566-590, SP, Brazil; jennifer.soares@usp.br (J.M.S.); kateblanco@ifsc.usp.br (K.C.B.); or bagnatovs@tamu.edu (V.S.B.); 2 Biomedical Engineering, Texas A&M University, 400 Bizzell St, College Station, TX 77843, USA; mitchellabonner@tamu.edu (M.B.); andrewrborsatto@tamu.edu (A.R.B.)

**Keywords:** antibiotic-resistant bacteria, *Staphylococcus aureus*, Fourier Transformation Infrared, minimum inhibitory concentration, machine learning algorithms

## Abstract

**Background/Objectives**: In recent work, we have demonstrated that principal component analysis (PCA) and Fourier Transformation Infrared (FTIR) spectra are powerful tools for analyzing the changes in microorganisms at the biomolecular level to detect changes in bacteria with resistance to antibiotics. Here biochemical structural changes in *Staphylococcus aureus* were analyzed over exposure time with the goal of identifying trends inside the samples that have been exposed to antibiotics for increasing amounts of time and developed resistance. **Methods**: All studied data was obtained from FTIR spectra of samples with induced antibiotic resistance to either Azithromycin, Oxacillin, or Trimethoprim/Sulfamethoxazole following the evolution of this development over four increasing antibiotic exposure periods. **Results**: The processing and data analysis with machine learning algorithms performed on this FTIR spectral database allowed for the identification of patterns across minimum inhibitory concentration (MIC) values associated with different exposure times and both clusters from hierarchical classification and PCA. **Conclusions**: The results enable the observation of resistance development pathways for the sake of knowing the present stage of resistance of a bacterial sample. This is carried out via machine learning methods for the purpose of faster and more effective infection treatment in healthcare settings.

## 1. Introduction

Surveillance data on cases of antimicrobial-resistant microorganisms has reported significant increases over the years [1]. The first metric for checking antibiotic susceptibility is determining the minimum inhibitory concentration (MIC), corresponding to the concentration capable of killing or inhibiting microbiological growth [2,3,4]. Antimicrobial resistance threatens the effective prevention and treatment of an ever-increasing range of infections caused by bacteria, parasites, viruses, and fungi [5,6,7,8,9,10,11,12]. Several mechanisms can lead to the development of resistance due to molecular and cellular modifications that can be acquired by genetic transfer via plasmid conjugation, bacteriophages [13,14,15,16], spontaneous mutations, or inappropriate exposure to antibiotics [14,15,16].

Most pathogenic microorganisms can develop resistance to at least some antimicrobial agents [14,15,16]. The main mechanisms of resistance are limiting uptake of a drug, modification of a drug target, inactivation of a drug, and active efflux of a drug [9,10,12,17]. These mechanisms may be native to microorganisms or acquired from other microorganisms [9,10,12,17]. For example, bacteria in water, soil, and air can acquire resistance following contact with resistant microorganisms [2,13,15,16,18,19]. When bacteria become resistant to antibiotics, treating the infection is often harder and more expensive [11,12,13,16,19,20,21,22,23]. Administering treatment without the risk of therapeutic failure is challenging, as patients often need immediate intervention before the results of antimicrobial tests are available. This involves a labor-intensive process where microorganisms must be cultured to identify them and assess their response to various classes of antimicrobials [5,6,8,9,10,11,23]. These practical limitations can lead to treatment failures and contribute to the development of antimicrobial resistance [1,20,24,25,26,27,28,29,30]. For this reason, optimizations of the process of identifying the antimicrobial susceptibility pattern are necessary.

A current challenge in antimicrobial resistance is the increasing resistance of bacteria to antibiotics and the search for ways to reduce it and improve the effectiveness of infection therapies [5,6,8,9,10,11,12,23]. Reports describe the emergence of microorganisms that are resistant to antibiotics and include surveillance studies collecting MIC values of different drugs [1]. An increase in MIC indicates increased resistance and, therefore, less susceptibility to specific antibiotics. In this study we introduced the use and application of the protocol regarding MIC values acquired for various times to the various antibiotics implemented by Soares et al. [31]. Here, we utilized statistical methods to analyze complex datasets of FTIR spectra for samples of *S. aureus* treated with three different kinds of antibiotics for four different exposure times across three biochemical windows. This was carried out by reducing dimensionality and identifying patterns in antibiotic resistance.

In this study, we built on the previous study which involved utilizing robust machine learning foundations to differentiate antimicrobial resistance [31,32,33,34,35]. FTIR spectral data provides a multidimensional database that can be analyzed by machine learning methods to consistently identify antibiotic susceptibility patterns in Gram-positive and Gram-negative microorganisms [31,32,33,34,35]. Here, we expand the analysis with additional machine learning tools and with the use of statistical tools and algorithms like principal component analysis (PCA) and hierarchical dendrograms with truncated clustering on FTIR spectra of methicillin-resistant *S. aureus* (MRSA). Samples were treated with Azithromycin (Azy), Oxacillin (Oxa), or Trimethoprim/Sulfamethoxazole (Trim) antibiotics, with exposure times of 0, 24, 72, and 120 h, creating a database of one thousand FTIR spectra.

Patterns were explored across FTIR data for the different antibiotics and the associated MIC values, which were measured in the samples analyzed for each antibiotic and time point [31]. To identify trends across the MRSA samples exposed to antibiotics, which generated resistance, we followed the development of the bacterial behavior when it was exposed to antibiotics for increasing amounts of time. Additionally, to analyze patterns in specific biomolecules, we isolated three smaller windows within the spectra that contained carbohydrates, proteins, and fatty acids. The acquisition of this large FTIR spectra dataset displaying resistance development over time allowed for the possibility of exploring and refining, via analysis with machine learning, the best course of action in cases of antibiotic resistance in real infections to improve treatment efficacy and speed. In this paper, the time-dependent development of resistance in *S. aureus* and its connection to MIC is explored in FTIR data utilizing hierarchical clustering and PCA.

## 2. Results

**Figure 1:**
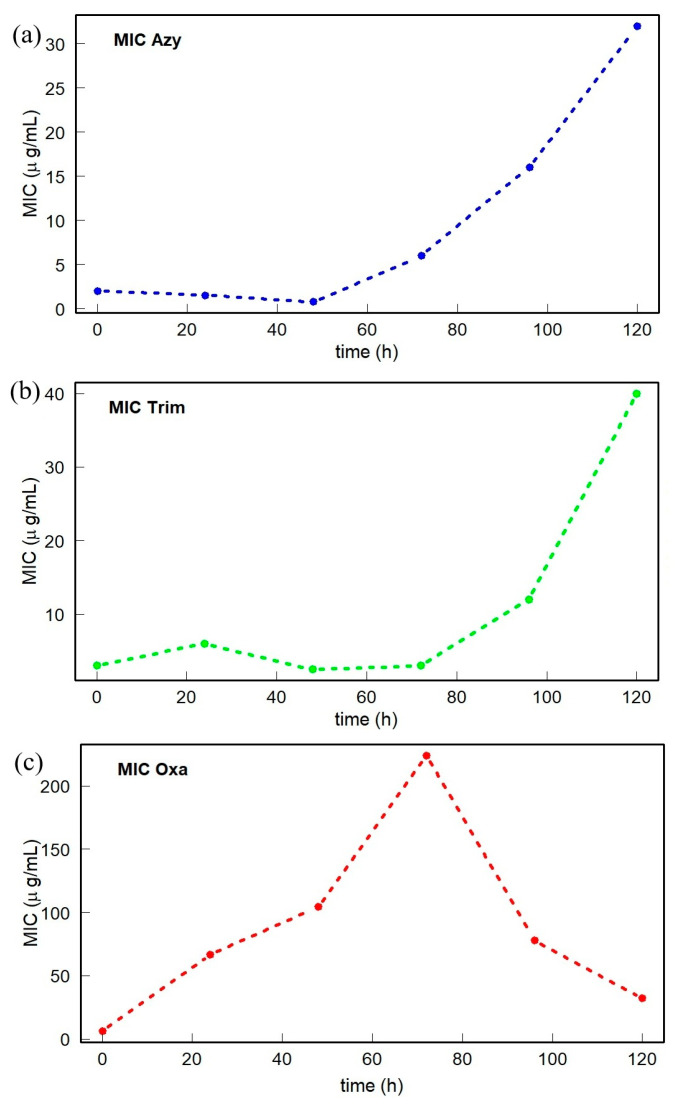
Representation of MIC values obtained for antibiotics (**a**) Azithromycin (Azy), (**b**) Trimethoprim/Sulfamethoxazole (Trim), and (**c**) Oxacillin (Oxa); data can be found in Table S1 in Supplemental Materials.

The *S. aureus* strain kept growing in the presence of antibiotics for 120 h, renewing the medium and the concentration of the antimicrobial every 12 h. In Figure 1, it is observed that MIC values vary over time. The continuous exposure of the strain to different antibiotics induces a less effective response of the antibiotic in the bacteria, as the MIC values at each new interval were higher, indicating an increase in antibiotic resistance. This behavior is notable for the antibiotics Azy and Trim. However, in the case of Oxa, the induction of resistance is observed to take place up to 72 h, after which the response to the antibiotic returns to approximately the initial level, so that the induced resistance has not been established in the bacterial culture.

**Figure 2:**
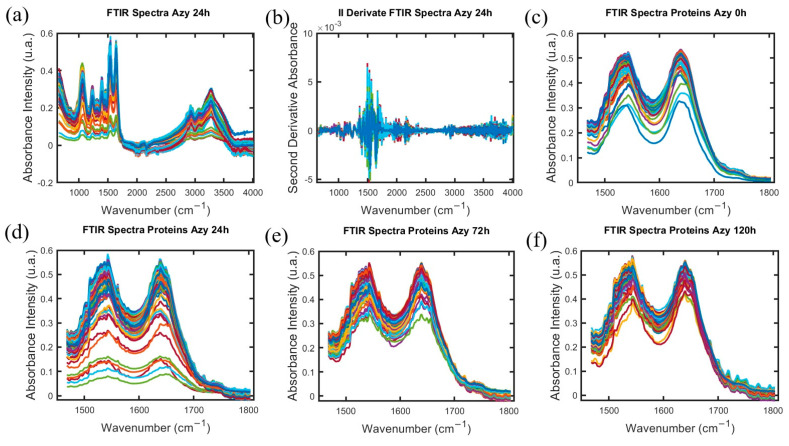
(**a**) FTIR spectra for one hundred samples of *S. aureus* with induced antibiotic resistance expose to Azithromycin for 24 h. (**b**) Result of applying the second derivate to FTIR spectra for one hundred samples of *S. aureus* with induced antibiotic resistance expose to Azithromycin for 24 h. (**c**–**f**) Visualization of normalized FTIR spectrum into proteins spectral regions between 1500 and 1800 cm^−1^ for (**c**) one hundred spectra of MRSA samples with no antibiotic exposure 0 h, (**d**) one hundred FTIR spectra of *S. aureus* with induced antibiotic resistance expose to Azithromycin for 24 h, (**e**) one hundred FTIR spectra of *S. aureus* with induced antibiotic resistance expose to Azithromycin for 72 h, (**f**) one hundred FTIR spectra of *S. aureus* with induced antibiotic resistance expose to Azithromycin for 120 h.

Given that the response to the antibiotic differs over the cultivation time of *S. aureus*, it is suggested that molecular and cellular changes are associated with this antibiotic-bacteria interaction. The molecular structure and mechanism of action of the antibiotics utilized are introduced in Table 1. Figure 2 shows the spectra obtained from the bacteria at different antibiotic cultivation intervals.

In Figure 2a we show the entire FTIR for all samples of *S. aureus* treated with Azithromycin (Azy) at 24 h. In Figure 2b we show the result after the second derivative process for the entire FTIR spectra. After both of these process the Normalization by maximum value of FTIR absorption intensity is performed in each one spectrum individually. Following the process to extract the window interval group, it is conforming the array of one hundred FTIR absorption spectra intensity for each exposition time to antibiotic with the same wavelength values. To illustrate the spectra obtained into protein window from FTIR spectra for the time exposition antibiotic Azithromycin (Azy) at 0 h 24 h, 72 h, 120 h the items Figure 2c–f are shown.

The FTIR spectrum identifies fingerprint regions of biomolecules in the bacterial cell structure, such as the carbohydrate spectral regions (950–1200 cm^−1^), fatty acids (2800–3100 cm^−1^), and proteins (1500–1800 cm^−1^). The processing of spectral data based on derivation and normalization (Figure 2) allows us to verify the differences of each biomolecule for each cultivation time (Figure 2c–f) of the bacteria in the presence of the different antibiotics. This process was developed utilizing tools in MATLAB [39].

The FTIR spectrum identifies fingerprint regions of biomolecules in the bacterial cell structure, such as carbohydrates, proteins, and fatty acids. The processing of spectral data based on derivation and normalization (Figure 2) allows us to verify the differences of each biomolecule for each cultivation time (Figure 2c–f) of the bacteria in the presence of the different antibiotics. This process was developed utilizing tools in MATLAB [39].

Hierarchical clustering methods and PCA (including PCA-center, or average PCA) machine learning tools were applied to the FTIR database of *S. aureus* with induced antibiotic resistance to Azy, Oxa, and Trim. The analysis of the FTIR spectra focused on specific biomolecular groups of carbohydrates between 950 and 1200 cm^−1^, fatty acids between 2800 and 3100 cm^−1^, and proteins between 1500 and 1800 cm^−1^. The connection between the spectral behavior of samples treated with the three antibiotics for four times was then studied. To develop the analyses, machine learning algorithms were implemented utilizing both supervised and unsupervised methods. A detailed description of these implemented methods is introduced in the materials and methods section. Machine learning algorithms have been developed in R [40,41,42,43,44,45,46,47,48] and Python (3.12.3) [49,50,51].

**Figure 3:**
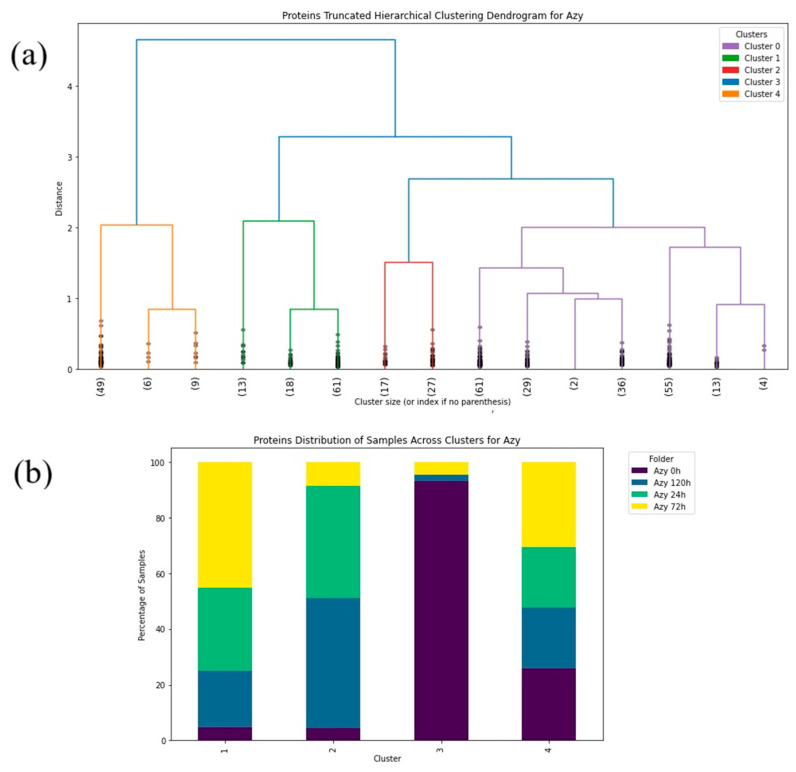
(**a**) Hierarchical dendrogram for the protein window with truncated clustering for all samples treated with Azithromycin at 0 h, 24 h, 72 h, 120 h. (**b**) Protein distribution of samples across each cluster for Azithromycin (Azy) at 0 h, 24 h, 72 h, 120 h.

**Figure 4:**
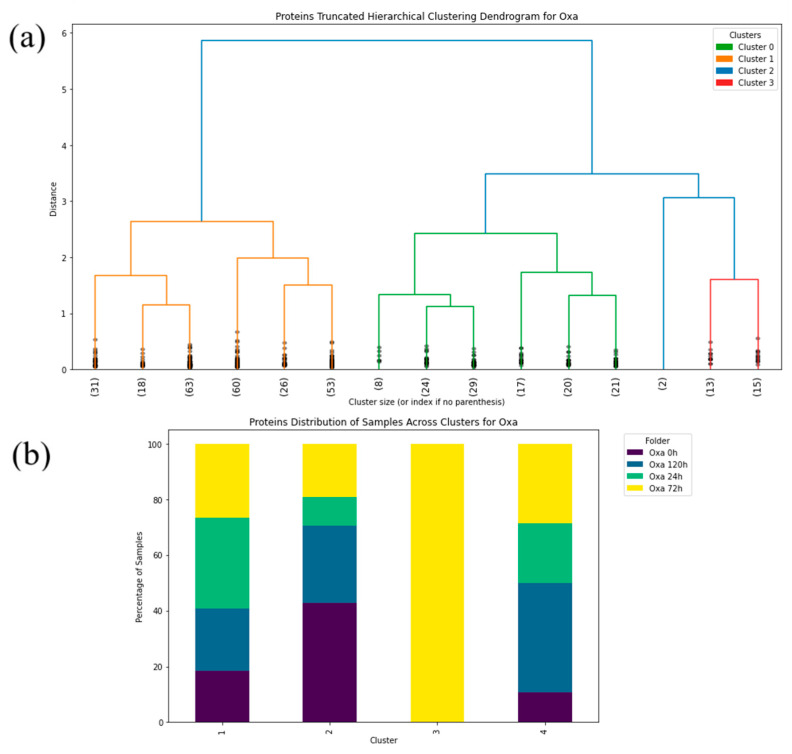
(**a**) Hierarchical dendrogram for the protein window with truncated clustering for all samples treated with Oxacillin at 0 h, 24 h, 72 h, 120 h. (**b**) Protein distribution of samples across each cluster for Oxacillin (Oxa) at 0 h, 24 h, 72 h, 120 h.

**Figure 5:**
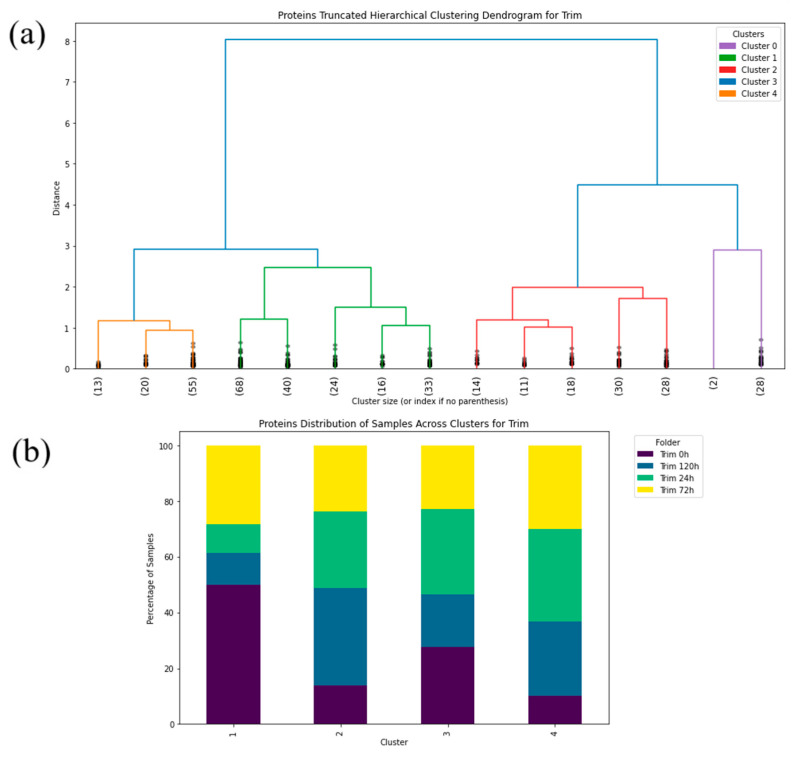
(**a**) Hierarchical dendrogram for the protein window with truncated clustering for all samples treated with Trimethoprim/Sulfamethoxazole at 0 h, 24 h, 72 h, 120 h. (**b**) Protein distribution of samples across each cluster for Trimethoprim/Sulfamethoxazole (Trim) at 0 h, 24 h, 72 h, 120 h.

**Figure 6:**
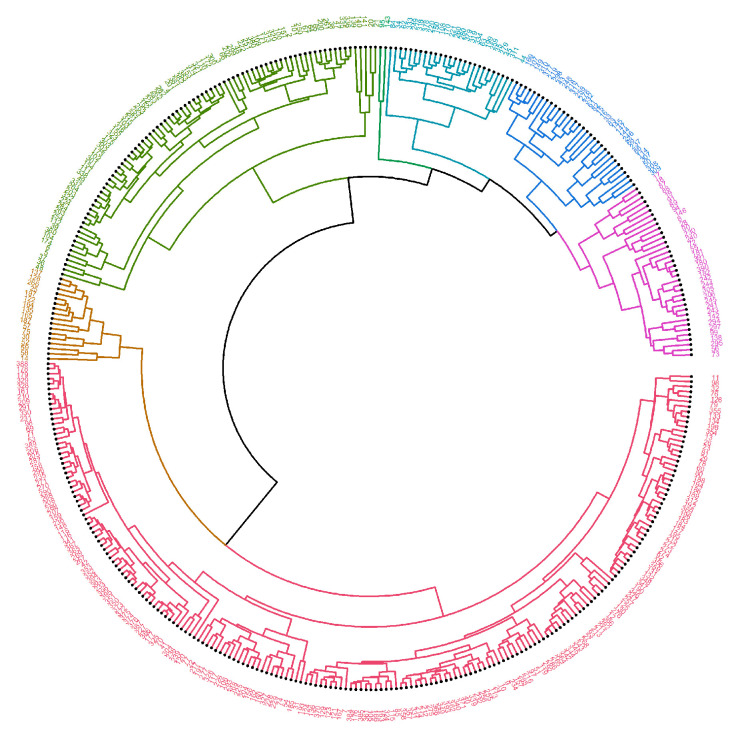
Circlized dendrogram for the protein region into the FTIR spectra for all samples of *S. aureus* treated with Azithromycin (Azy) at 24 h, 72 h, 120 h and the average MRSA samples with no antibiotic exposure (0 h).

**Figure 7:**
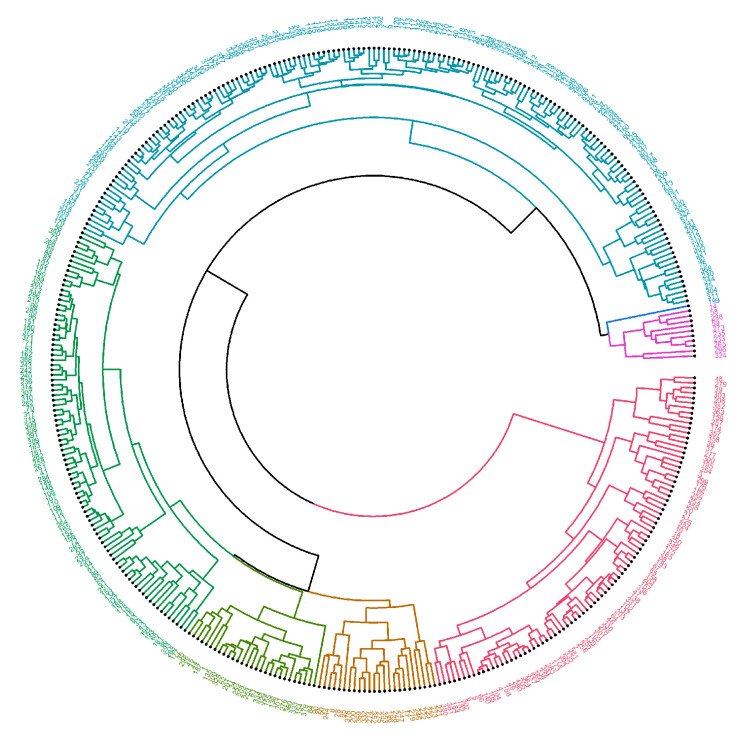
Circlized dendrogram for the protein region into the FTIR spectra for all samples of *S. aureus* treated with Oxacillin (Oxa) at 24 h, 72 h, 120 h and the average MRSA samples with no antibiotic exposure (0 h).

**Figure 8:**
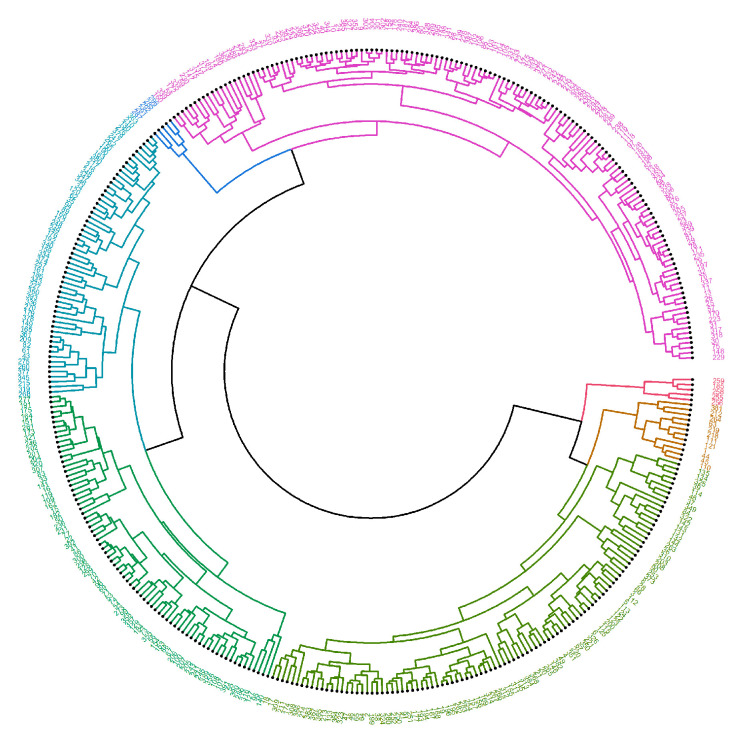
Circlized dendrogram for the protein region into the FTIR spectra for all samples of *S. aureus* treated with Trimethoprim/Sulfamethoxazole (Trim) at 24 h, 72 h, 120 h and the average MRSA samples with no antibiotic exposure (0 h).

Hierarchical clustering was conducted utilizing the “Ward” linkage method, which minimizes the sum of squares differences within clusters [52]. This method is particularly effective for spectroscopic data since it tends to highlight the natural grouping based on spectral similarity. The truncated dendrogram method was applied to focus on the major cluster formations of the FTIR spectra in the protein (Figure 3, Figure 4, Figure 5, Figure 6, Figure 7 and Figure 8), carbohydrate (Figures S1–S3, Supplementary Materials) and fatty acid (Figures S4–S6, Supplementary Materials) biochemical windows.

Circlized dendrograms were obtained to capture the classifications of all FTIR spectra with more than four main branches. In the initial calculation steps, four main branches were expected due to the four acquisition times present in the data. However, due to dynamic biochemical properties and antibiotic resistance mechanisms present, the dendrogram exhibits seven main representative branches, with a significant mix of time points present in each branch for proteins (Figure 4, Figure 5, Figure 6, Figure 7 and Figure 8), carbohydrates (Figures S7–S9, Supplementary Materials), and fatty acids (Figures S10–S12, Supplementary Materials). To easily identify the variety of time points represented in each cluster (branch), the samples were labeled such that numbers 1–100 correspond to samples obtained at 0 h, 101–199 to those obtained at 24 h, 200–298 to those obtained at 72 h, and 299–397 to those obtained at 120 h. Clusters are shown in different colors into circlized dendrogram representation in Figure 4, Figure 5, Figure 6, Figure 7 and Figure 8 and Figures S7–S12 in Supplementary Materials.

The data analyzed with PCA methods generated plots with the first two principal components that correspond to the largest amounts of variance in the data in proteins (Figures S9–S11), carbohydrates (Figures S13–S15, Supplementary Materials), and fatty acids (Figures S16–S18, Supplementary Materials) FTIR spectra windows. As a result, PCA can reveal patterns across resistances for the three different implemented antibiotics. Here, the groups are referred to as 0 h, 24 h, 72 h, 120 h, and iB (the control, non-methicillin resistant sample). The PCA calculation and the data visualization were carried out in R [40,41,42,43,44,45,46,47,48] and Python [49,50,51].

## 3. Discussion

The plots in Figure 1 displaying MIC changes over time for all three antibiotics indicate the need to be able to distinguish between different exposure times for the sake of improved treatments. For Azy and Trim, the MIC grows at an increasing rate with exposure time, but for Oxa the MIC peaks at 72 h and then nearly declines to initial values as time goes on. This difference is significant and suggests that different behaviors might be discovered in spectral analysis. These different behaviors of resistance development necessitate different time windows be considered for the study of the evolution of induced antibiotic resistance.

**Figure 9:**
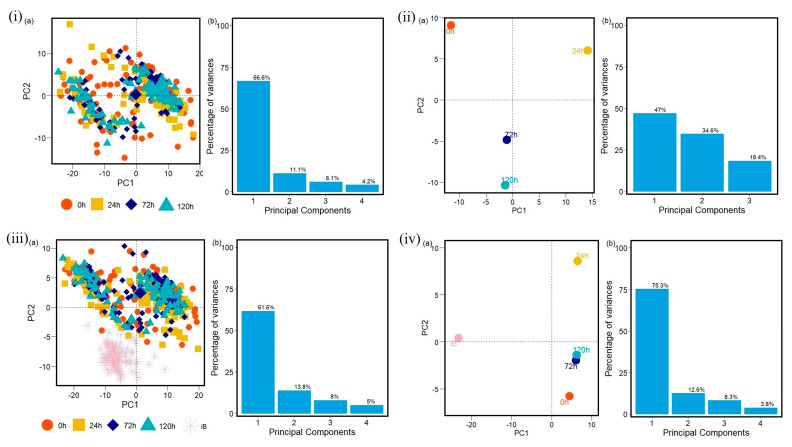
(**i**) Principal component analysis PCA (a) with variances in (b) and (**ii**) PCA-center calculations results to protein region into the FTIR spectra for MRSA samples with no antibiotic exposure (0 h), and for all samples of MRSA treated with Azithromycin (Azy) at 24 h, 72 h, 120 h. (**iii**,**iv**) PCA and PCA-center calculation results obtained to protein region into the FTIR spectra for MRSA samples with no antibiotic exposure (0 h), for all samples of MRSA treated with Azithromycin (Azy) at 24 h, 72 h, 120 h, and for all non-methicillin-resistant *S. aureus* samples (iB). Each data group analyzed into protein region at 0 h, 24 h, 72 h, 120 h and iB contains one hundred FTIR spectra samples.

**Figure 10:**
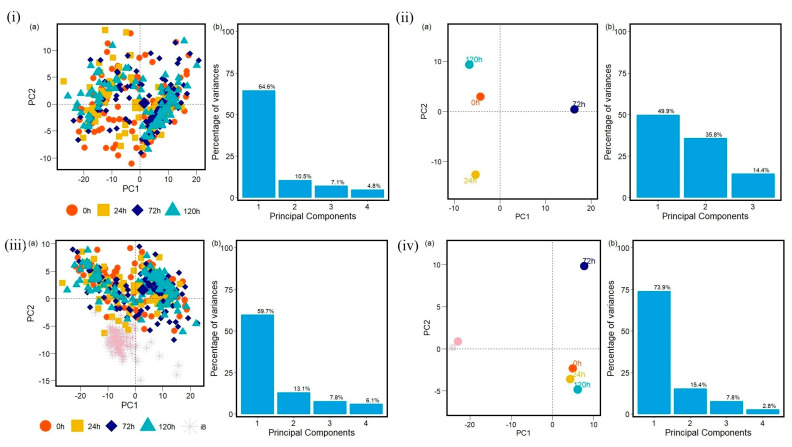
(**i**) Principal component analysis PCA (a) with variances in (b) and (**ii**) PCA-center calculations results to protein region into the FTIR spectra for MRSA samples with no antibiotic exposure (0 h), and for all samples of MRSA treated with Oxacillin (Oxa) at 24 h, 72 h, 120 h. (**iii**,**iv**) PCA and PCA-center calculation results obtained to protein region into the FTIR spectra for MRSA samples with no antibiotic exposure (0 h), for all samples of MRSA treated with Oxacillin (Oxa) at 24 h, 72 h, 120 h, and for all non-methicillin-resistant *S. aureus* samples (iB). Each data group analyzed into protein region at 0 h, 24 h, 72 h, 120 h and iB contains one hundred FTIR spectra samples.

**Figure 11:**
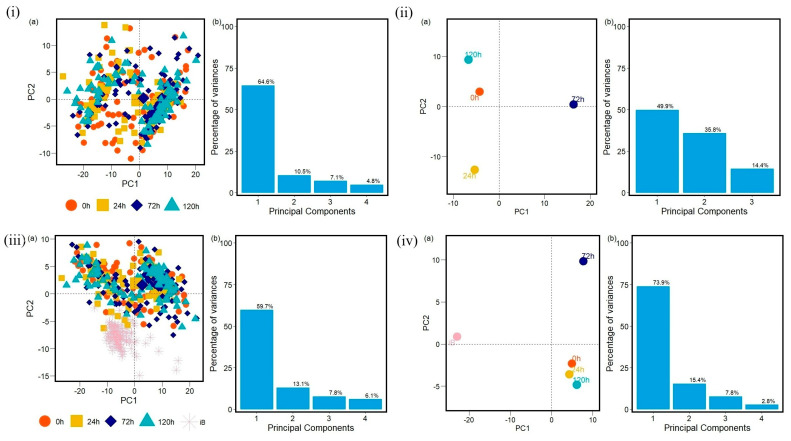
(**i**) Principal component analysis PCA (a) with variances in (b) and (**ii**) PCA-center calculations results to protein region into the FTIR spectra for MRSA samples with no antibiotic exposure (0 h), and for all samples of MRSA treated with Trimethoprim/Sulfamethoxazole (Trim) at 24 h, 72 h, 120 h. (**iii**,**iv**) PCA and PCA-center calculation results obtained to protein region into the FTIR spectra for MRSA samples with no antibiotic exposure (0 h), for all samples of MRSA treated Trimethoprim/Sulfamethoxazole (Trim) at 24 h, 72 h, 120 h, and for all non-methicillin-resistant *S. aureus* samples (iB). Each data group analyzed into protein region at 0 h, 24 h, 72 h, 120 h and iB contains one hundred FTIR spectra samples.

The control strain, which is susceptible to the antibiotics evaluated, differs from all of the PCA data obtained from the resistant strains. Furthermore, the PCA data obtained demonstrates that the influence of each antibiotic affects each biomolecule differently. During the first 24 h, the interaction with the antibiotics causes cellular stress that leads to changes in the vibrational modes of the biomolecules [53,54], as evidenced by the distinct quadrant of the 24 h data for most of the biomolecules from the different antibiotic cultures. However, the biological response is not homogeneous for all cells within a population under stress conditions. There will be a portion of the population that will be more susceptible and another that will be resistant. This explains why hierarchical clusterization results in more than four clusters, with each containing a mix of samples from various time points in the branch of the dendrograms. This behavior is then also visible in the PCA spatial classifications (Figure 3, Figure 4, Figure 5, Figure 6, Figure 7, Figure 8, Figure 9(i–iii), Figure 10(i–iii) and Figure 11(i–iii), respectively). With an increase in the MIC value (Figure S1 and Table S1 in Supplementary Materials), the responses’ heterogeneity increases, leading to a large dispersion of data in the PCA plots (Figure 9, Figure 10 and Figure 11). Consequently, over time, the bacterial culture can solidify with resistance to certain classes of antibiotics or can increase its susceptibility, which the average PCA visualization shows with some centers of mass for 120 h close to those for 0 h.

Though it is not fully explored in this analysis, the difference over time indicated in the principal components suggests that a value could be calculated and plotted over time such that it would mirror the MIC behavior. This would allow resistance to be predicted in a sample from its FTIR spectrum without having to carry out time-consuming MIC experiments. The most prominent obstacle in this pursuit is the clear presence of overlap across time points as demonstrated in the dendrogram results with window intervals for proteins, carbohydrates, and fatty acids. An increase in exposure time does not provide a unified increase in resistance across all samples. This poses a significant challenge when it comes to experimentally identifying observable and consistent changes in structure.

Hierarchical clustering methodology involves building clusters by measuring the similarities between data. The hierarchy of clusters is shown by combining and splitting groups at different levels of similarity. The chosen clustering algorithms, therefore, need to be efficient [55]. The methodology implemented in the current study is a new processing tool that helps to improve the methodology previously used on samples with antibiotic susceptibility studied in a single period [32,35]. This allows for a study of *S. aureus* samples with various levels of antibiotic susceptibility that takes into account the evolution over time with machine learning algorithms.

Truncated dendrogram results offer a simplified and insightful view into the most dominant data groupings that emerged in the time ranges of the analysis to identify the intrinsic array of the samples with induced antibiotic resistance to proteins (Figure 3, Figure 4 and Figure 5), carbohydrates (Figures S1–S3, Supplementary Materials) and fatty acids (Figures S4–S6, Supplementary Materials). This approach demonstrated the complex nature of the spectral data being studied and the relationships of its clusters, which indicates the need for a classification based on multiple features to effectively cluster the data into resistance categories. This statistical procedure implemented into the algorithm has provided a clear picture of the dynamics and the hierarchical structure of the FTIR spectral data. Cluster percentage tables obtained by *S. aureus* in individual time (0 h, 24 h, 72 h, and 120 h) to the antibiotics are show in Supplementary Materials (Tables S2–S10).

The circlized dendrograms show that the antibiotic resistance mechanism is somewhat unique in each sample. The samples have reacted to the surrounding medium that contains different antibiotics for different amounts of time. The variation is due to dynamic biochemical properties and antibiotic resistance mechanisms developed in the various time periods. Furthermore, these behaviors likely also appear, and create this kind of variety, in exposure times outside of the four explored in this study. This behavior is perceived in proteins (Figure 4, Figure 5, Figure 6, Figure 7 and Figure 8), carbohydrates (Figures S7–S9, Supplementary Materials), and fatty acids (Figures S10–S12, Supplementary Materials).

Results obtained from the truncated and circlized dendrograms demonstrate strong agreement with the PCA classification of the samples. The overlapping of samples with various exposure time is due to the fact that microorganisms can have different activation periods and react to specific antibiotics with different interacting mechanisms (Table S1, Supplementary Materials). It displays a high dependence between microorganisms and antibiotics due to the biochemical reactions with the surrounding medium. Additionally, it is to be expected that the entire sample does not have the same response to the antibiotic at the same time. It is interesting to note that this biochemical behavior is reflected in all our results obtained from the supervised and unsupervised machine learning algorithms used here.

All results obtained here display a high dependence on time. The results show a temporal correlation between the development of resistance and changes in the FTIR spectra, identified in the PCA and machine learning analyses. The overlapping of groups in the PCA plots is related to the idea that as resistance develops over time, it becomes more difficult to distinguish between these levels of induced resistance. There is, however, a prominent difference between samples with no exposure and those with any amount of exposure.

When the first and second principal components are plotted for all of the samples with no averaging, it becomes almost impossible to distinguish between any of the MRSA samples visually, though the control sample stands out as a clear separate group when included. These patterns generally hold true for the Oxa and Trim samples as well, though the variation between the first and later time points is mainly present in the windows for carbohydrates and fatty acids, and less so for proteins (Figures S7(i,iii)–S9(i,iii) and S13(i,iii)–S18(i,iii) in Supplementary Materials, respectively). The first principal component in the protein windows generally explains a much smaller percentage of the variance than those of the other two windows across all three antibiotics. The bulk of the explained variance, therefore, for proteins is distributed across more of the principal components. This indicates that relationships are more difficult to capture in this window with two principal components and might help to explain this observation of less observable variation.

The PCA-center plots for the Azy samples clearly show that for each of the three biochemical windows (carbohydrates, fatty acids, and proteins), the average MRSA sample with no antibiotic exposure (0 h) varies noticeably from the later time points (24 h, 72 h, 120 h) (Figure 9ii). Additionally, if the average control sample, or non-methicillin-resistant *S. aureus* sample is included (iB), it shows significant variation from the four average MRSA samples (Figure 9iv). The later time points generally vary less from each other and do not display a clear pattern of difference, though some variation is still present.

Notably, since the control sample (iB) varied significantly from the MRSA samples, which then showed internal variation, it can be concluded that the FTIR spectra change enough with the accumulation of resistances such that classification is still possible with the presence of many overlapping resistances to different antibiotics. Additionally, the fact that significant amounts of variance are explained by principal components beyond the first two in the biomolecular windows indicates the potential to explore the present relationships in PCA in more than two dimensions. This might prove helpful in the exploration of molecular changes over time with antibiotic exposure.

## 4. Materials and Methods

### 4.1. Samples Preparation and FTIR Spectra Acquisition

#### 4.1.1. Resistance Induction

*Staphylococcus aureus* (NIST 0023) was cultured in brain heart infusion agar, from which colonies were collected for preparation of the inoculum in Mueller Hinton Cation Adjusted (MHCA) medium, standardized at 108 colony forming units (CFU/mL) from optical density at 600 nm. Antibiotic concentrations corresponding to 1.5 times the Minimum Inhibitory Concentration (MIC) of Azy, Trim, and Oxa were added to the inoculum. Every 12 h, the culture was centrifuged for 10 min, 3000 rpm, and resuspended in a new medium containing antibiotic. In this interval, samples were collected and plated to determine the MIC.

#### 4.1.2. Minimum Inhibitory Concentration

In a 96-well plate, the antibiotic concentrations were diluted 2-fold in MHCA medium, and then the bacterial culture collected at different cultivation intervals was added, ensuring a maximum concentration in the wells of 106 CFU/mL. After 24 h, the MIC was determined with the aid of resazurin.

#### 4.1.3. Fourier Transformation Infrared (FTIR) Spectra Acquisition

Susceptibility to antibiotic samples was prepared. FTIR spectrum samples were acquired following the protocol by Soares et al. [33,34] for all utilized samples of *S. aureus*. From the plated samples, the colonies were kept growing at 37 °C for 24–48 h so that the colonies reached sufficient sizes to be deposited on the crystal in the FTIR equipment by Attenuated Total Reflection (ATR) on the Agilent Cary 630 FTIR Spectrometer^®^ instrument (Agilent Technologies, Billerica, MA, USA) in the wavelength range of (650–4000) cm^−1^. The dry sample was scanned 250 times, with a resolution of 4 cm^−1^. All of these samples together made up one thousand and one hundred FTIR spectra.

### 4.2. Methodology and Machine Learning Algorithms

#### 4.2.1. Machine Learning—Data Processing

Code written in Python was utilized to read and process the raw FTIR data. Initially, in order to highlight variation, the second derivative was taken and a Savitzky–Golay smoothing filter was applied. The data was then normalized using min-max normalization before the spectral windows for carbohydrates (900–1200 cm^−1^), proteins (1500–1800 cm^−1^), and fatty acids (2800–3100 cm^−1^) were isolated. The derivatives, normalization, and window isolation for visualizations were performed manually in MATLAB R2022b [48] and Python (3.12.3) [45], while the smoothing was performed with functions from the SciPy open-source library in Python [45,46].

Details about the supervised and unsupervised learning machine learning algorithms implemented in this study can be consulted in our previous research work about identification of antibiotic resistance in microorganism [31,32,33,34,35], into methodology sections “FTIR Absorption Spectrum of *S. aureus* Acquisition and Data Process in MATLAB”, “Supervised/Unsupervised Machine Learning Algorithms Applied to Spectrum Analysis” and “Machine learning algorithms” in [32] and sections “FTIR Spectra Database Analysis Process Overview” and “Machine Learning Algorithms into the methodology” section in [35].

#### 4.2.2. Machine Learning—Hierarchical Clustering

Given a set of FTIR spectra data to be clustered, the distance matrix or similarity matrix is created. Here, the Euclidean distance and the Ward linkage methods were implemented into the hierarchical clustering routine section of the machine learning algorithm developed to carry out the agglomeration process. In Ward’s method, successive clustering steps are used to minimize the increase in error in the sum of squares at each step.

A function was created to cluster the data based on their intensity patterns using hierarchical clustering method. This method builds a hierarchy of clusters by iteratively merging the closest pairs of clusters, starting with individual data points as their own cluster. This function was used in exploratory data analysis to identify natural groupings or patterns in spectral data across multiple samples and within specific time groups (0 h, 24 h, 72 h, and 120 h). By visualizing the dendrogram, the data structure was better understood and informed decisions on the number of clusters representing the grouping data can be made. Additionally, another function was created in which truncated dendrograms were implemented with the “lastp” truncated mode and the “Ward variance minimization algorithm” to join different clusters. The truncated dendrograms only display the last p merged clusters of the hierarchical clustering process, which simplifies the dendrogram to help focus on the significant cluster formations at the final stages of the hierarchy.

The circlized dendrogram process begins by taking each spectrum as a single independent cluster. It then applies Euclidean distance as a metric to measure similarity between the points that constitute each spectrum, finding the two closest FTIR spectra in the analyzed biochemical window and combining them into a cluster. It continues combining the clusters based on similarities between the spectrum values until there is only one cluster containing all of the spectra. The method uses agglomerative clustering steps that follow a bottom-up approach.

#### 4.2.3. Machine Learning—Principal Component Analysis

PCA and PCA-center data analyses take into account the entire FTIR spectra sample with induced antibiotic resistance for each biochemical group of interest. All of the data from each biomolecular window (carbohydrates, fatty acids and proteins) for each antibiotic are transformed from a large number of spectrum values into a set of two principal components. The most significant variations are found within the spectrum data for each window for each kind of antibiotic used on *S. aureus* for four exposure times. This allows us to interpret and visualize data arrays across many variables. This means that, for example, the carbohydrate spectral regions were analyzed (950–1200 cm^−1^) for one hundred spectra of MRSA samples with no antibiotic exposure (0 h), one hundred FTIR spectra samples of *S. aureus* with 24 h of exposure, one hundred FTIR spectra samples of *S. aureus* with 72 h of exposure, and one hundred FTIR spectra samples of *S. aureus* with 120 h of exposure to antibiotic.

Four hundred samples for each antibiotic were, therefore, then studied in each correspondent analysis for the carbohydrate windows. With the group at 0 h being the control group for due to the lack of exposure to antibiotics, and the groups called 24 h, 72 h, 120 h corresponding to samples of MRSA exposed to antibiotics for these time periods. The iB data contains one hundred spectra from non-methicillin-resistant *S. aureus* and is added for comparison. Wherever the iB data is not shown, the PCA calculation was carried out on only the four hundred MRSA spectra. Where the iB data is present, this was a new PCA calculation on five hundred spectra including all MRSA spectra and the iB spectra.

The same methodology was applied to the FTIR spectral regions for fatty acids (2800–3100 cm^−1^), and proteins (1500–1800 cm^−1^). In the main article, results of PCA for the protein windows for all samples treated with the antibiotics Azithromycin, Oxacillin and Trimethoprim/Sulfamethoxazole at 0 h, 24 h, 72 h, and 120 h are shown. The results for carbohydrates and fatty acids are shown in the Supplementary Materials of this article (Figures S13–S18).

Code written in R and Python were applied to perform principal component analysis (PCA). This was carried out utilizing the processed (second derivative, normalized, and smoothed) data for samples in every exposure time group for each window. PCA was performed with functions from the Scikit-learn open-source library in Python [45,47]. The data reduction process is performed in our analysis of FTIR spectra with PCA in R Project for Statistical Computing (4.2.3) [40,41,42,43,44,45,46,47,48], Matlab R2022b [39] and Python (3.12.3) [45,47].

## 5. Conclusions

The MIC value is an important metric of resistance to different antibiotics in bacteria. The MIC values obtained with continuous exposure of the strain to the antibiotics Azy and Trim at each new interval were higher, indicating an increase in antibiotic resistance. In the case of Oxa, however, the induction of resistance is observed up to 72 h, after which the response to the antibiotic returns to approximately the initial level, so that the induced resistance has not been established in the bacterial culture.

The different behavior of MIC values in response to the antibiotics (Figure 1) is connected to the specific biochemical reaction of the samples in each time period. This mechanism is reflected in the hierarchical clustering of the samples as well as the spatial distribution of PCA-centers for the FTIR spectra of the samples (Figure 9, Figure 10 and Figure 11). A high congruence is demonstrated between the intrinsic biochemical antibiotic resistance mechanism of the studied samples and the arrangement of the samples in the agglomeration in the truncated dendrogram, the branches of the circlized dendrogram, and the spatial distribution in the PCA space.

If the results from this analysis were to be utilized for the purpose of selecting a treatment plan, it would be important to determine what is considered “resistant” and “non-resistant.” While the appropriate binary classifications are not explored here, the need for them is significant and demonstrates the importance of distinguishing between the different antibiotic exposure ranges of time analyzed in our samples. This significant application sets the stage for more studies regarding the behavior of antibiotic reaction mechanisms over time in microorganisms.

Though it is not fully explored in this analysis, the difference over time indicated in the principal components suggests that a value could be calculated and plotted over time such that it would mirror the MIC behavior. This is challenging, however, as an increase in exposure time does not provide a unified increase in antibiotic resistance across all samples. The data demonstrates that this failure of bacteria to develop resistance uniformly can mean that a sample exposed to antibiotics for a long period of time can have a biomolecular composition that is more similar to samples with no exposure than to samples with the same long exposure time that happened to develop resistance.

Additionally, the fact that significant amounts of variance are explained by principal components beyond the first two in the biomolecular windows indicates the potential to explore the present relationships in PCA in more than two dimensions. This might prove helpful in the exploration of molecular changes over time with antibiotic exposure.

## Figures and Tables

**Table 1:** Molecular structure and mechanism of action for the antibiotics used in this study.

Antibiotic	Chemical Structure	Properties
Azithromycin (Azy) C_38_H_72_N_2_O_12_ [36]	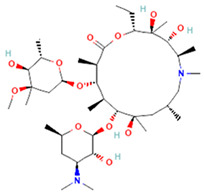	In order to replicate, bacteria require a specific process of protein synthesis enabled by ribosomal proteins. Azithromycin binds to the 23S rRNA of the bacterial 50S ribosomal subunit. It stops bacterial protein synthesis by inhibiting the transpeptidation/translocation step of protein synthesis and by inhibiting the assembly of the 50S ribosomal subunit. Azithromycin is highly stable at a low pH, giving it a longer serum half-life and increasing its concentrations in tissues compared to erythromycin [36].
Oxacillin (Oxa) C_19_H_19_N_3_O_5_S [37]	** 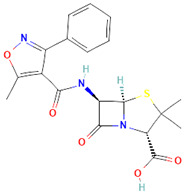 **	By binding to specific penicillin-binding proteins (PBPs) located inside the bacterial cell wall, Oxacillin inhibits the third and last stage of bacterial cell wall synthesis. Cell lysis is then mediated by bacterial cell wall autolytic enzymes such as autolysins; it is possible that Oxacillin interferes with an autolysin inhibitor [37].
Trimethoprim/Sulfamethoxazole (Trim) C_14_H_18_N_4_O_3_ [38]	** 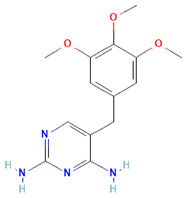 **	Trimethoprim is a reversible inhibitor of dihydrofolate reductase, one of the principal enzymes catalyzing the formation of tetrahydrofolic acid (THF) from dihydrofolic acid (DHF). Tetrahydrofolic acid is necessary for the biosynthesis of bacterial nucleic acids and proteins and ultimately for continued bacterial survival—inhibiting its synthesis, which then results in bactericidal activity. Trimethoprim is often given in combination with sulfamethoxazole, which inhibits the preceding step in bacterial protein synthesis. Given together, sulfamethoxazole and trimethoprim inhibit two consecutive steps in the biosynthesis of bacterial nucleic acids and proteins [38].

## Data Availability

The data presented in this study are available on request from the corresponding authors.

## Supplementary Materials

The following supporting information can be downloaded at: https://www.mdpi.com/article/10.3390/antibiotics14070729/s1; Table S1. MIC values obtained by *S. aureus* in individual time and to the three-antibiotic implemented into the study. Table S2. Proteins Cluster percentage table obtained by *S. aureus* in individual time (0 h, 24 h, 72 h, and 120 h) and Azithromycin (Azy) exposure. Table S3. Protein Cluster percentage table obtained by *S. aureus* in individual time (0 h, 24 h, 72 h, and 120 h), and Oxacillin (Oxa) exposure. Table S4. Proteins Cluster percentage table obtained by *S. aureus* in individual time (0 h, 24 h, 72 h, and 120 h) and Trimethoprim/ Sulfamethoxazole (Trim) exposure. Table S5. Carbohydrates Cluster percentage table obtained by *S. aureus* in individual time (0 h, 24 h, 72 h, and 120 h) and Azithromycin (Azy) exposure. Table S6. Carbohydrates Cluster percentage table obtained by *S. aureus* in individual time (0 h, 24 h, 72 h, and 120 h), and Oxacillin (Oxa) exposure. Table S7. Carbohydrates Cluster percentage table obtained by *S. aureus* in individual time (0 h, 24 h, 72 h, and 120 h) and Trimethoprim/ Sulfamethoxazole (Trim) exposure. Table S8. Fatty Acids Cluster percentage table obtained by *S. aureus* in individual time (0 h, 24 h, 72 h, and 120 h) and Azithromycin (Azy) exposure. Table S9. Fatty Acids Cluster percentage table obtained by *S. aureus* in individual time (0 h, 24 h, 72 h, and 120 h) and Oxacillin (Oxa) exposure. Table S10. Fatty Acids Cluster percentage table obtained by *S. aureus* in individual time (0 h, 24 h, 72 h, and 120 h) and Trimethoprim/ Sulfamethoxazole (Trim) exposure. Figure S1. (a) Carbohydrates Hierarchical dendrograms with truncated clustering to the entire sample for Azithromycin (Azy) at 0 h, 24 h, 72 h, 120 h. (b) Carbohydrates Distribution of Samples Across each Cluster for Azithromycin (Azy) at 0 h, 24 h, 72 h, 120 h. Figure S2. (a) Carbohydrates Hierarchical dendrograms with truncated clustering to the entire sample for Oxacillin (Oxa) at 0 h, 24 h, 72 h, 120 h. (b) Carbohydrates Distribution of Samples Across each Cluster for Oxacillin (Oxa) at 0 h, 24 h, 72 h, 120 h. Figure S3. (a) Carbohydrates Hierarchical dendrograms with truncated clustering to the entire sample for Trimethoprim/ Sulfamethoxazole (Trim) at 0 h, 24 h, 72 h, 120 h. (b) Carbohydrates Distribution of Samples Across each Cluster for Trimethoprim/ Sulfamethoxazole (Trim) at 0 h, 24 h, 72 h, 120 h. Figure S4. (a) Fatty Acids Hierarchical dendrograms with truncated clustering to the entire sample for Azithromycin (Azy) at 0 h, 24 h, 72 h, 120 h. (b) Fatty Acids Distribution of Samples Across each Cluster for Azithromycin (Azy) at 0 h, 24 h, 72 h, 120 h. Figure S5. (a) Fatty Acids Hierarchical dendrograms with truncated clustering to the entire sample for Oxacillin (Oxa) at 0 h, 24 h, 72 h, 120 h. (b) Fatty Acids Distribution of Samples Across each Cluster for Oxacillin (Oxa) at 0 h, 24 h, 72 h, 120 h. Figure S6. Fatty Acids Hierarchical dendrograms with truncated clustering to the entire sample for Trimethoprim/ Sulfamethoxazole (Trim) at 0 h, 24 h, 72 h, 120 h. (b) Fatty Acids Distribution of Samples Across each Cluster for Trimethoprim/ Sulfamethoxazole (Trim) at 0 h, 24 h, 72 h, 120 h. Figure S7. Circlized dendrogram results for carbohydrates region into the FTIR spectra for all samples of *S. aureus* treated with Azithromycin (Azy) at 24 h, 72 h, 120 h and the average MRSA samples with no antibiotic exposure (0 h). Figure S8. Circlized dendrogram results for carbohydrates region into the FTIR spectra for all samples of *S. aureus* treated with Oxacillin (Oxa) at 24 h, 72 h, 120 h and the average MRSA samples with no antibiotic exposure (0 h). Figure S9. Circlized dendrogram results for carbohydrates region into the FTIR spectra for all samples of *S. aureus* treated with Trimethoprim/Sulfamethoxazole (Trim) at 24 h, 72 h, 120 h and the average MRSA samples with no antibiotic exposure (0 h). Figure S10. Circlized dendrogram results for fatty acids region into the FTIR spectra for all samples of *S. aureus* treated with Azithromycin (Azy) at 24 h, 72 h, 120 h and the average MRSA samples with no antibiotic exposure (0 h). Figure S11. Circlized dendrogram results for fatty acids region into the FTIR spectra for all samples of *S. aureus* treated with Oxacillin (Oxa) at 24 h, 72 h, 120 h and the average MRSA samples with no antibiotic exposure (0 h). Figure S12. Circlized dendrogram results for fatty acids region into the FTIR spectra for all samples of *S. aureus* treated with Trimethoprim/Sulfamethoxazole (Trim) at 24 h, 72 h, 120 h and the average MRSA samples with no antibiotic exposure (0 h). Figure S13. (i) Principal component analysis PCA (a) with variances in (b) and (ii) PCA-center calculations results to carbohydrates region into the FTIR spectra for MRSA samples with no antibiotic exposure (0 h), and for all samples of MRSA treated with Azithromycin (Azy) at 24h, 72h, 120h. (iii,iv) PCA and PCA-center calculation results obtained to carbohydrates region into the FTIR spectra for MRSA samples with no antibiotic exposure (0 h), for all samples of MRSA treated with Azithromycin (Azy) at 24 h, 72 h, 120 h, and for all non-methicillin-resistant *S. aureus* samples (iB). Each data group analyzed into carbohydrates region at 0 h, 24 h, 72 h, 120 h and iB contains one hundred FTIR spectra samples. Figure S14. (i) Principal component analysis PCA (a) with variances in (b) and (ii) PCA-center calculations results to carbohydrates region into the FTIR spectra for MRSA samples with no antibiotic exposure (0 h), and for all samples of MRSA treated with Oxacillin (Oxa) at 24 h, 72 h, 120 h. (iii,iv) PCA and PCA-center calculation results obtained to carbohydrates region into the FTIR spectra for MRSA samples with no antibiotic exposure (0 h), for all samples of MRSA treated with Oxacillin (Oxa) at 24 h, 72 h, 120 h, and for all non-methicillin-resistant *S. aureus* samples (iB). Each data group analyzed into carbohydrates region at 0 h, 24 h, 72 h, 120 h and iB contains one hundred FTIR spectra samples. Figure S15. (i) Principal component analysis PCA (a) with variances in (b) and (ii) PCA-center calculations results to carbohydrates region into the FTIR spectra for MRSA samples with no antibiotic exposure (0 h), and for all samples of MRSA treated with Trimethoprim/ Sulfamethoxazole (Trim) at 24 h, 72 h, 120 h. (iii,iv) PCA and PCA-center calculation results obtained to carbohydrates region into the FTIR spectra for MRSA samples with no antibiotic exposure (0 h), for all samples of MRSA treated Trimethoprim/ Sulfamethoxazole (Trim) at 24 h, 72 h, 120 h, and for all non-methicillin-resistant *S. aureus* samples (iB). Each data group analyzed into carbohydrates region at 0 h, 24 h, 72 h, 120 h and iB contains one hundred FTIR spectra samples. Figure S16. (i) Principal component analysis PCA (a) with variances in (b) and (ii) PCA-center calculations results to fatty acids region into the FTIR spectra for MRSA samples with no antibiotic exposure (0 h), and for all samples of MRSA treated with Azithromycin (Azy) at 24 h, 72 h, 120 h. (iii,iv) PCA and PCA-center calculation results obtained to fatty acids region into the FTIR spectra for MRSA samples with no antibiotic exposure (0 h), for all samples of MRSA treated with Azithromycin (Azy) at 24 h, 72 h, 120 h, and for all non-methicillin-resistant *S. aureus* samples (iB). Each data group analyzed into fatty acids region at 0 h, 24 h, 72 h, 120 h and iB contains one hundred FTIR spectra samples. Figure S17. (i) Principal component analysis PCA (a) with variances in (b) and (ii) PCA-center calculations results to fatty acids region into the FTIR spectra for MRSA samples with no antibiotic exposure (0 h), and for all samples of MRSA treated with Oxacillin (Oxa) at 24 h, 72 h, 120 h. (iii,iv) PCA and PCA-center calculation results obtained to fatty acids region into the FTIR spectra for MRSA samples with no antibiotic exposure (0 h), for all samples of MRSA treated with Oxacillin (Oxa) at 24 h, 72 h, 120 h, and for all non-methicillin-resistant *S. aureus* samples (iB). Each data group analyzed into fatty acids region at 0 h, 24 h, 72 h, 120 h and iB contains one hundred FTIR spectra samples. Figure S18. (i) Principal component analysis PCA (a) with variances in (b) and (ii) PCA-center calculations results to fatty acids region into the FTIR spectra for MRSA samples with no antibiotic exposure (0 h), and for all samples of MRSA treated with Trimethoprim/ Sulfamethoxazole (Trim) at 24 h, 72 h, 120 h. (iii,iv) PCA and PCA-center calculation results obtained to fatty acids region into the FTIR spectra for MRSA samples with no antibiotic exposure (0 h), for all samples of MRSA treated Trimethoprim/ Sulfamethoxazole (Trim) at 24 h, 72 h, 120 h, and for all non-methicillin-resistant *S. aureus* samples (iB). Each data group analyzed into fatty acids region at 0 h, 24 h, 72 h, 120 h and iB contains one hundred FTIR spectra samples.
