# Supplementary material for: Time Evolution of Bacterial Resistance Observed with Principal Component Analysis
Source: Antibiotics (Basel). 2025 Jul 20;14(7):729. doi: 10.3390/antibiotics14070729 (PMC12291692; doi:10.3390/antibiotics14070729)
Supplement: Supplementary file 1 [file antibiotics-14-00729-s001.zip › antibiotics-3737716-supplementary.pdf]

antibiotics

Supplementary material

Time Evolution of Bacterial Resistance Observed with Principal Component Analysis

Claudia P. Barrera Patiño [1,2*], Mitchell Bonner [2], Andrew Ramos Borsatto [2], Jennifer M. Soares [1,2], Kate C. Blanco [1], Vanderlei S. Bagnato [1,2]

1 Sao Carlos Institute of Physics (IFSC), University of Sao Paulo (USP), Sao Carlos 13566-590, SP, Brazil; jennifer.soares@usp.br (J.M.S.) https://orcid.org/0000-0002-2978-7076; kateblanco@ifsc.usp.br (K.C.B.) https://orcid.org/0000-0003-0361-9725; vander@ifsc.usp.br (V.S.B.) https://orcid.org/0000-0003-4833-239X

2 Biomedical Engineering, Texas A&M University, 400 Bizzell St, College Station, TX 77843, USA; mitchellabonner@tamu.edu (M.B.) https://orcid.org/0009-0001-3119-9986; andrewrborsatto@tamu.edu (A.R.B.) https://orcid.org/0009-0005-3852-4648; bagnatovs@tamu.edu (V.S.B.) https://orcid.org/0000-0003-4833-239X

* Correspondence: cpbarrerapatino@tamu.edu or cpbarrerap@ifsc.usp.br (C.P.B.P.) https://orcid.org/0000-0002-3233-1109

1. Minimum Inhibitory Concentration (MIC) values associated with different exposure times

In table 1, it is possible observe that MIC values vary in time, perhaps it can be interpretated like levels of susceptibility or resistance to antibiotic implemented here are dependent on the defense mechanism of the bacterial strain to applied antibiotic. This is interesting due to the MIC is the concentration of an antibiotic needed to inhibit bacterial growth. Then the change with the time involved establishing the treatments in a safe way. In this procedure the PCA interrelationship with MIC value can bring the tool to identify and help to establish with accuracy what the time of antibiotic resistance bacteria are and help to establish the treatments.

Table S1. MIC values obtained by *S. aureus* in individual time and to the three-antibiotic implemented into the study.

Time (h)	MIC (µg/mL)		
	Azithromycin	Oxacillin	Trimethoprim/ Sulfamethoxazole
**0**	2	6	3
**24**	1.5	66.5	6
**48**	0.75	104.25	2.5
**2**	6	224.125	3
**96**	16	78	12
**120**	32	32	40

Academic Editor: Firstname Lastname

Received: date
Revised: date
Accepted: date
Published: date

Citation: To be added by editorial staff during production.

Copyright: © 2025 by the authors. Submitted for possible open access publication under the terms and conditions of the Creative Commons Attribution (CC BY) license (https://creativecommons.org/licenses/by/4.0/).

2. Clustering and Hierarchical Dendrograms Results

Table S2. Proteins Cluster percentage table obtained by *S. aureus* in individual time (0 h, 24 h, 72 h, and 120 h) and Azithromycin (Azy) exposure.

Cluster	Antibiotic Exposure	Percentage
1	Azy 0h	4.69
1	Azy 120h	20.31
1	Azy 24h	29.69
1	Azy 72h	45.31
2	Azy 0h	4.35
2	Azy 120h	46.74
2	Azy 24h	40.22
2	Azy 72h	8.7
3	Azy 0h	93.18
3	Azy 120h	2.27
3	Azy 72h	4.55
4	Azy 0h	26
4	Azy 120h	21.5
4	Azy 24h	22
4	Azy 72h	30.5

Table S3. Protein Cluster percentage table obtained by *S. aureus* in individual time (0 h, 24 h, 72 h, and 120 h), and Oxacillin (Oxa) exposure.

Cluster	Antibiotic Exposure	Percentage
1	Oxa 0h	18.33
1	Oxa 120h	22.31
1	Oxa 24h	32.67
1	Oxa 72h	26.69
2	Oxa 0h	42.86
2	Oxa 120h	27.73
2	Oxa 24h	10.08
2	Oxa 72h	19.33
3	Oxa 72h	100
4	Oxa 0h	10.71
4	Oxa 120h	39.29
4	Oxa 24h	21.43
4	Oxa 72h	28.57

Table S4. Proteins Cluster percentage table obtained by *S. aureus* in individual time (0 h, 24 h, 72 h, and 120 h) and Trimethoprim/ Sulfamethoxazole (Trim) exposure.

Cluster	Antibiotic Exposure	Percentage
1	Trim 0h	50
1	Trim 120h	11.36
1	Trim 24h	10.23
1	Trim 72h	28.41
2	Trim 0h	13.81
2	Trim 120h	34.81
2	Trim 24h	27.62
2	Trim 72h	23.76
3	Trim 0h	27.72
3	Trim 120h	18.81

3	Trim 24h	30.69
3	Trim 72h	22.77
4	Trim 0h	10
4	Trim 120h	26.67
4	Trim 24h	33.33
4	Trim 72h	30

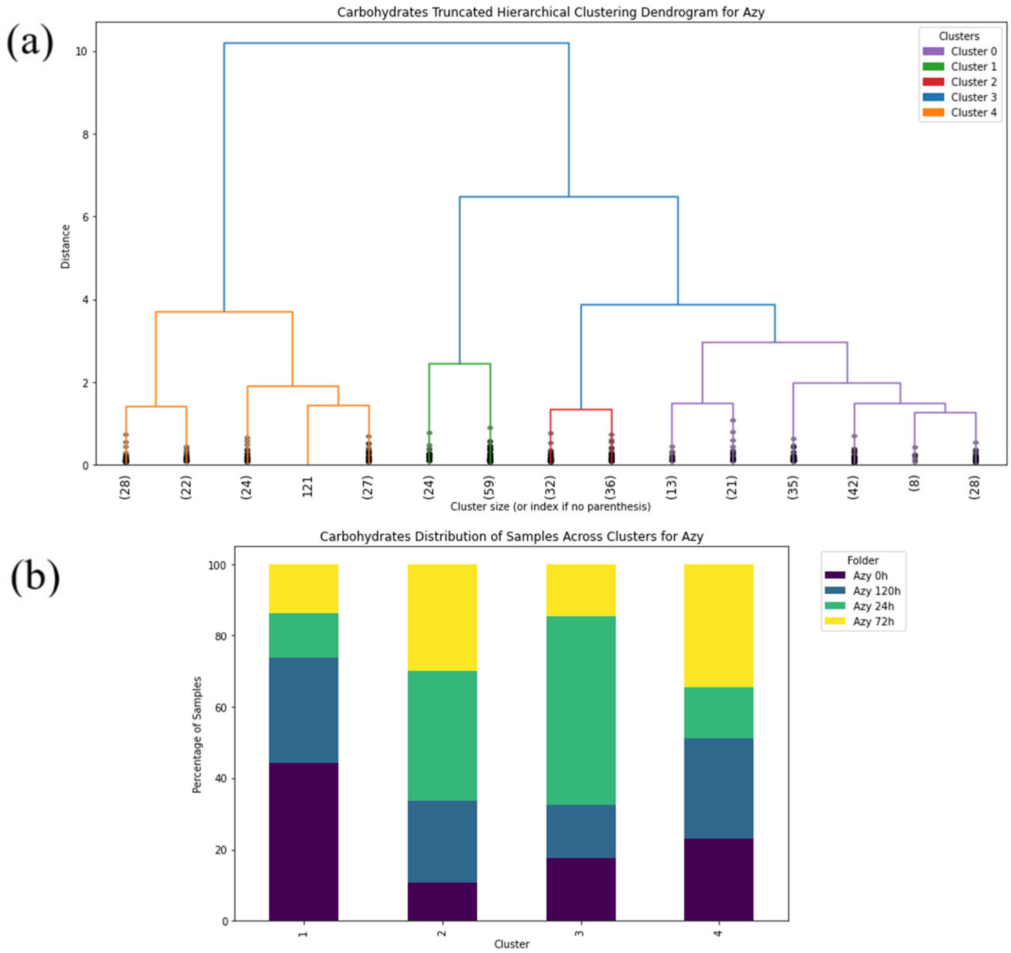

Figure S1. (a) Carbohydrates Hierarchical dendrograms with truncated clustering to the entire sample for Azithromycin (Azy) at 0 h, 24 h, 72 h, 120 h. (b) Carbohydrates Distribution of Samples Across each Cluster for Azithromycin (Azy) at 0 h, 24 h, 72 h, 120 h.

Table S5. Carbohydrates Cluster percentage table obtained by *S. aureus* in individual time (0 h, 24 h, 72 h, and 120 h) and Azithromycin (Azy) exposure.

Cluster	Antibiotic Exposure	Percentage
1	Azy 0h	44.12
1	Azy 120h	29.41
1	Azy 24h	12.75
1	Azy 72h	13.73
2	Azy 0h	10.84
2	Azy 120h	22.89
2	Azy 24h	36.14
2	Azy 72h	30.12
3	Azy 0h	17.65
3	Azy 120h	14.71
3	Azy 24h	52.94

3	Azy 72h	14.71
4	Azy 0h	23.13
4	Azy 120h	27.89
4	Azy 24h	14.29
4	Azy 72h	34.69

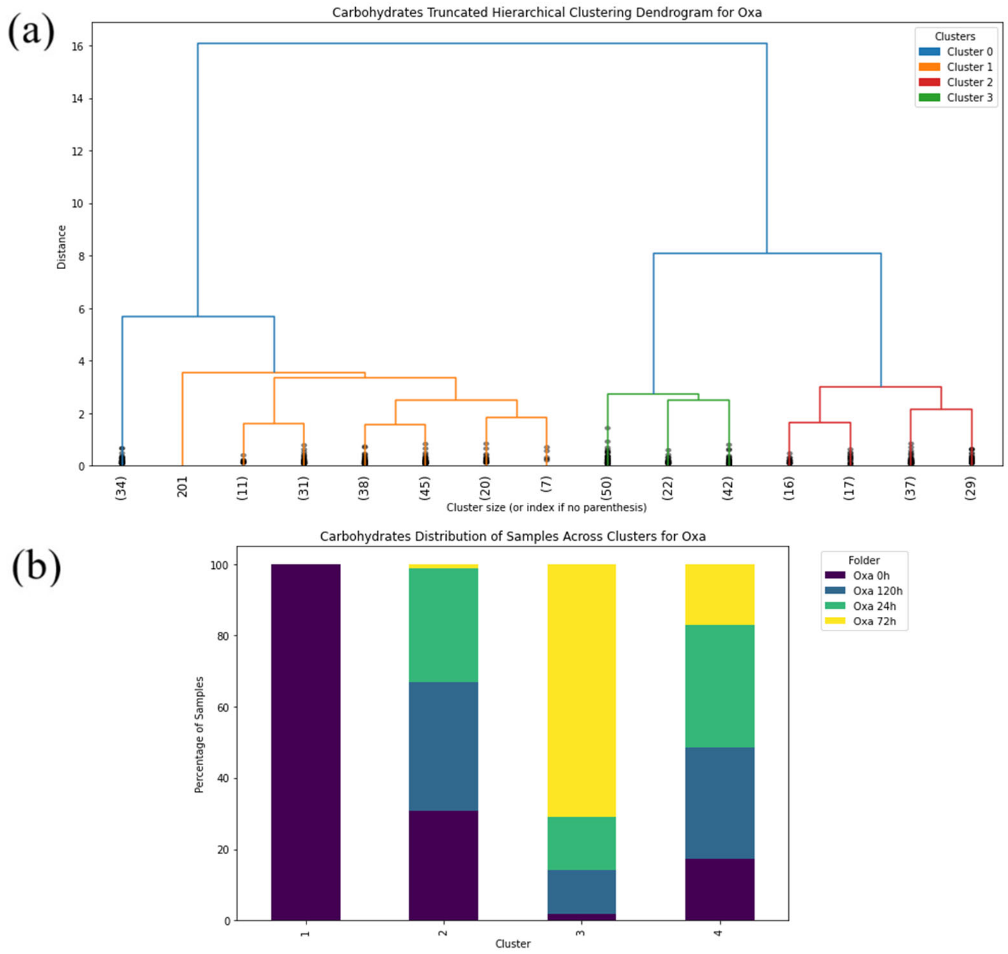

Figure S2. (a) Carbohydrates Hierarchical dendrograms with truncated clustering to the entire sample for Oxacillin (Oxa) at 0 h, 24 h, 72 h, 120 h. (b) Carbohydrates Distribution of Samples Across each Cluster for Oxacillin (Oxa) at 0 h, 24 h, 72 h, 120 h.

Table S6. Carbohydrates Cluster percentage table obtained by *S. aureus* in individual time (0h, 24h, 72h, and 120h), and Oxacillin (Oxa) exposure.

Cluster	Antibiotic Exposure	Percentage
1	Oxa 0h	100
2	Oxa 0h	30.72
2	Oxa 120h	35.95
2	Oxa 24h	32.03
2	Oxa 72h	1.31
3	Oxa 0h	1.75
3	Oxa 120h	12.28
3	Oxa 24h	14.91
3	Oxa 72h	71.05
4	Oxa 0h	17.17
4	Oxa 120h	31.31
4	Oxa 24h	34.34

4	Oxa 72h	17.17

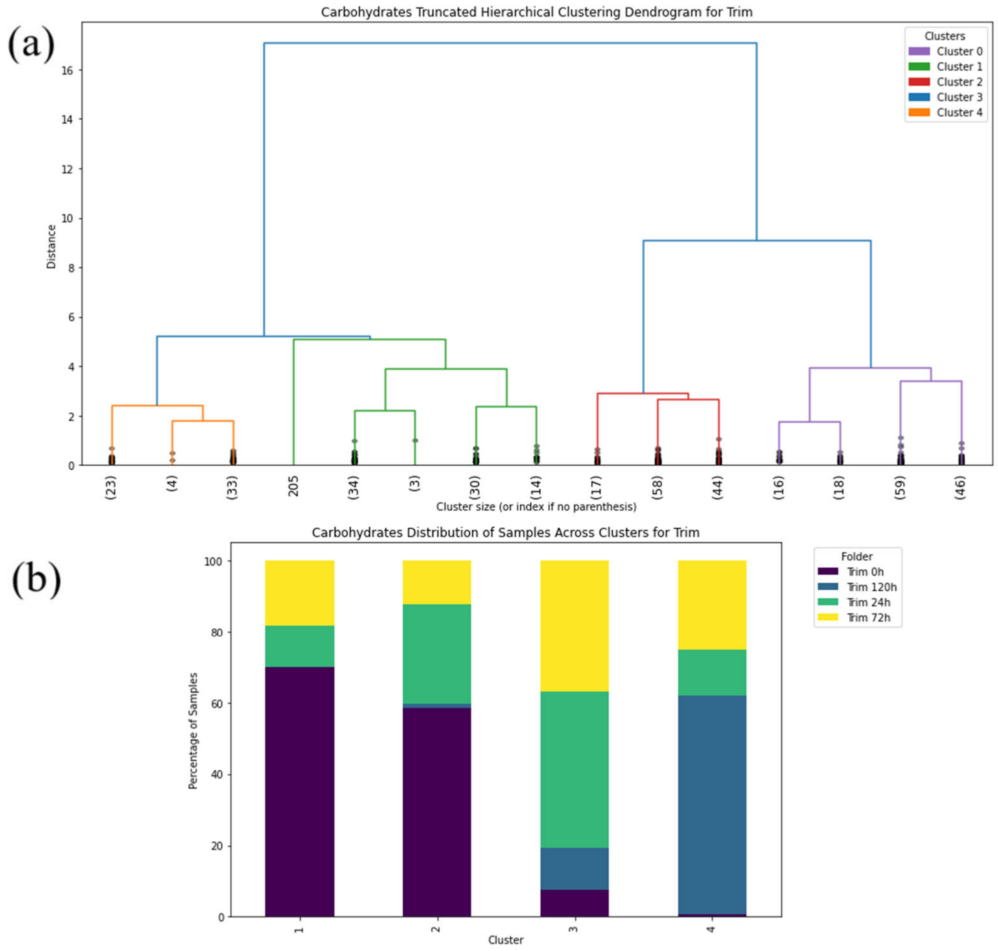

Figure S3. (a) Carbohydrates Hierarchical dendrograms with truncated clustering to the entire sample for Trimethoprim/ Sulfamethoxazole (Trim) at 0 h, 24 h, 72 h, 120 h. (b) Carbohydrates Distribution of Samples Across each Cluster for Trimethoprim/ Sulfamethoxazole (Trim) at 0 h, 24 h, 72 h, 120 h.

Table S7. Carbohydrates Cluster percentage table obtained by *S. aureus* in individual time (0 h, 24 h, 72 h, and 120 h) and Trimethoprim/ Sulfamethoxazole (Trim) exposure.

Cluster	Antibiotic Exposure	Percentage
1	Trim 0h	70
1	Trim 24h	11.67
1	Trim 72h	18.33
2	Trim 0h	58.54
2	Trim 120h	1.22
2	Trim 24h	28.05
2	Trim 72h	12.2
3	Trim 0h	7.56
3	Trim 120h	11.76
3	Trim 24h	43.7
3	Trim 72h	36.97
4	Trim 0h	0.72
4	Trim 120h	61.15
4	Trim 24h	12.95
4	Trim 72h	25.18

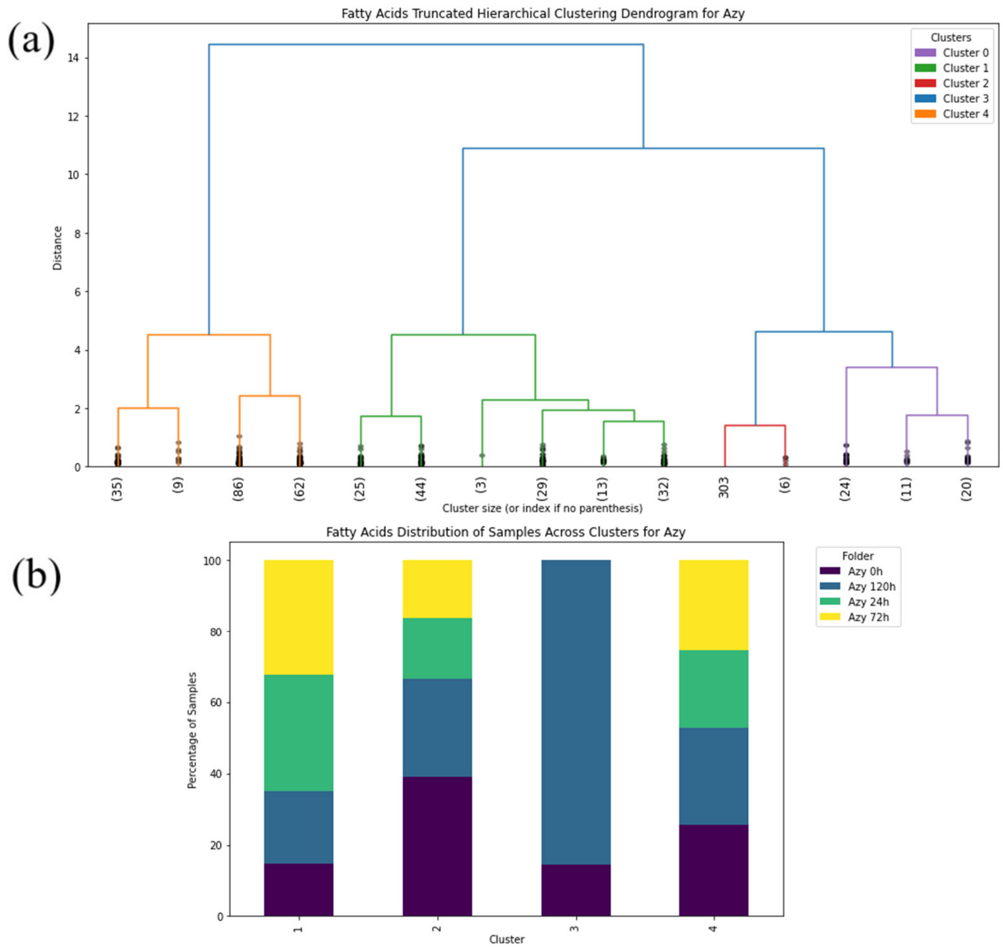

Figure S4. (a) Fatty Acids Hierarchical dendrograms with truncated clustering to the entire sample for Azithromycin (Azy) at 0 h, 24 h, 72 h, 120 h. (b) Fatty Acids Distribution of Samples Across each Cluster for Azithromycin (Azy) at 0 h, 24 h, 72 h, 120 h.

Table S8. Fatty Acids Cluster percentage table obtained by *S. aureus* in individual time (0 h, 24 h, 72 h, and 120 h) and Azithromycin (Azy) exposure.

Cluster	Antibiotic Exposure	Percentage
1	Azy 0h	14.58
1	Azy 120h	20.31
1	Azy 24h	32.81
1	Azy 72h	32.29
2	Azy 0h	39.04
2	Azy 120h	27.4
2	Azy 24h	17.12
2	Azy 72h	16.44
3	Azy 0h	14.29
3	Azy 120h	85.71
4	Azy 0h	25.45
4	Azy 120h	27.27
4	Azy 24h	21.82
4	Azy 72h	25.45

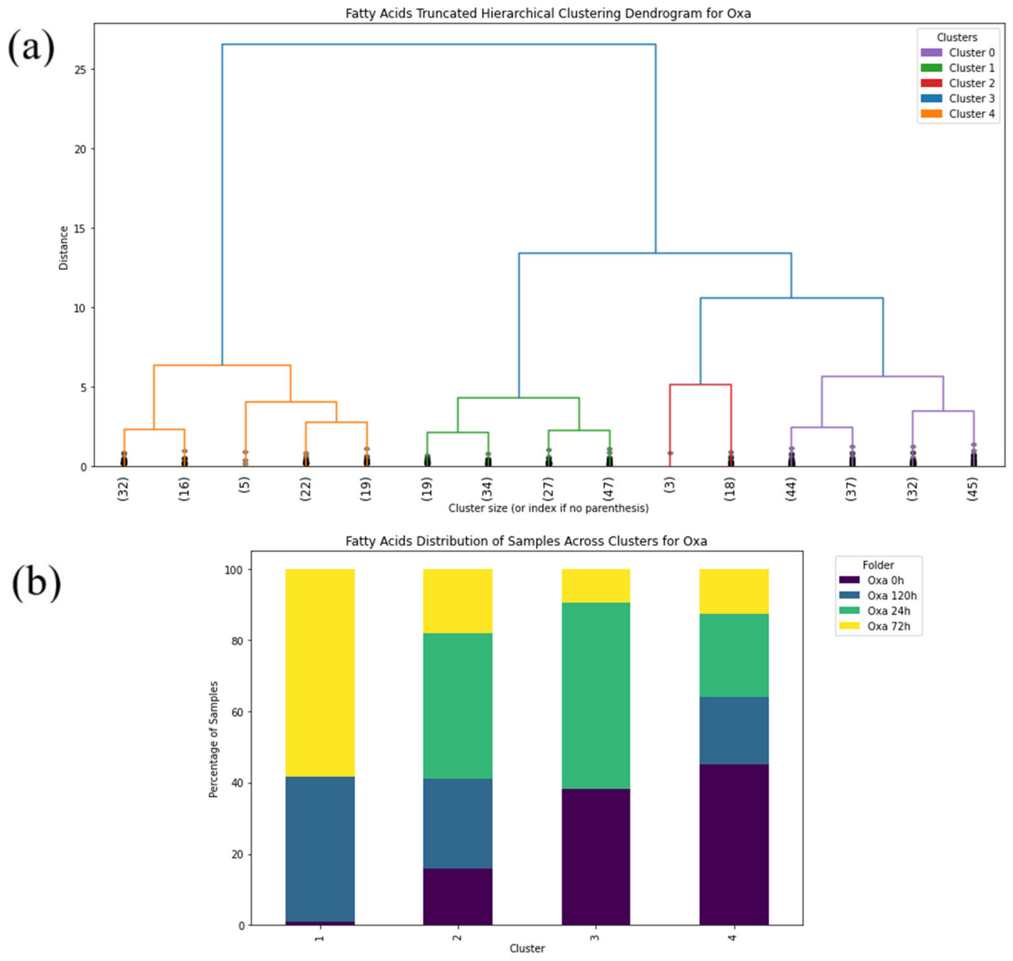

Figure S5. (a) Fatty Acids Hierarchical dendrograms with truncated clustering to the entire sample for Oxacillin (Oxa) at 0 h, 24 h, 72 h, 120 h. (b) Fatty Acids Distribution of Samples Across each Cluster for Oxacillin (Oxa) at 0 h, 24 h, 72 h, 120 h.

Table S9. Fatty Acids Cluster percentage table obtained by *S. aureus* in individual time (0 h, 24 h, 72 h, and 120 h) and Oxacillin (Oxa) exposure.

Cluster	Antibiotic Exposure	Percentage
1	Oxa 0h	1.06
1	Oxa 120h	40.43
1	Oxa 72h	58.51
2	Oxa 0h	15.75
2	Oxa 120h	25.2
2	Oxa 24h	40.94
2	Oxa 72h	18.11
3	Oxa 0h	38.1
3	Oxa 24h	52.38
3	Oxa 72h	9.52
4	Oxa 0h	44.94
4	Oxa 120h	18.99
4	Azy 24h	21.82
4	Azy 72h	25.45

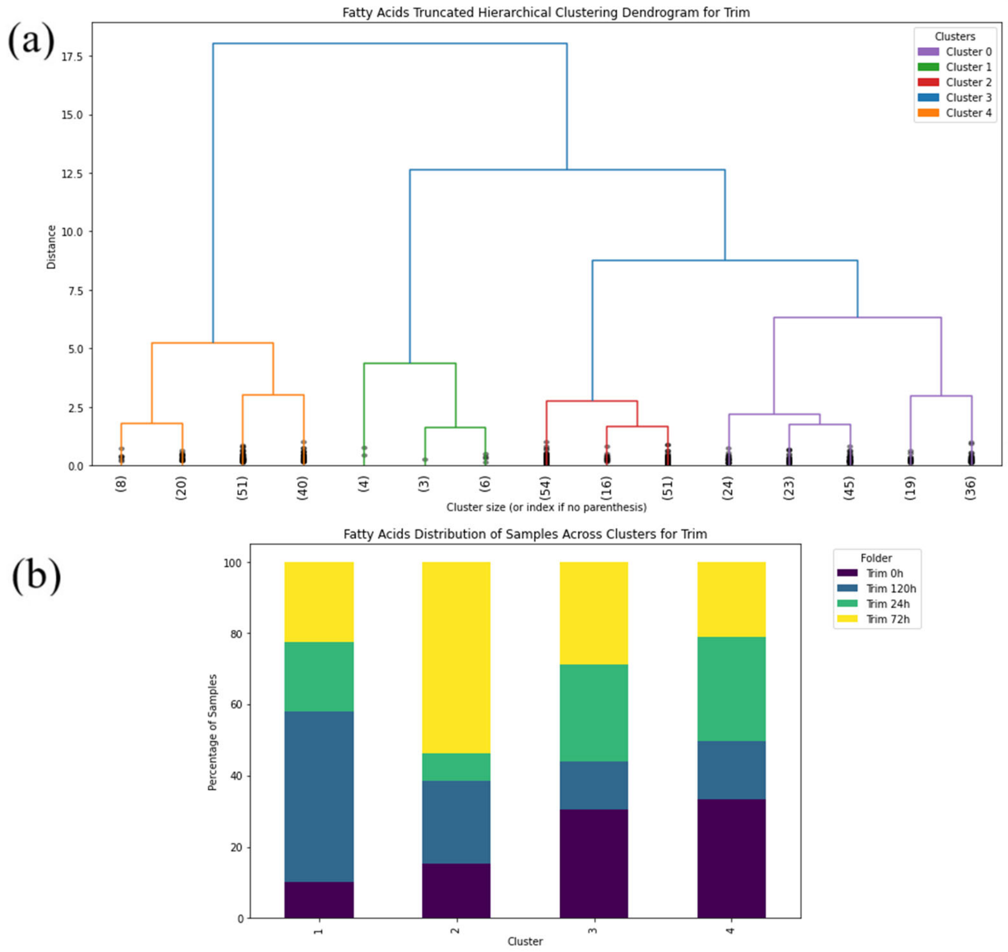

Figure S6. Fatty Acids Hierarchical dendrograms with truncated clustering to the entire sample for Trimethoprim/ Sulfamethoxazole (Trim) at 0 h, 24 h, 72 h, 120 h. (b) Fatty Acids Distribution of Samples Across each Cluster for Trimethoprim/ Sulfamethoxazole (Trim) at 0 h, 24 h, 72 h, 120 h.

Table S10. Fatty Acids Cluster percentage table obtained by *S. aureus* in individual time (0 h, 24 h, 72 h, and 120 h) and Trimethoprim/ Sulfamethoxazole (Trim) exposure.

Cluster	Antibiotic Exposure	Percentage
1	Trim 0h	10.08
1	Trim 120h	47.9
1	Trim 24h	19.33
1	Trim 72h	22.69
2	Trim 0h	15.38
2	Trim 120h	23.08
2	Trim 24h	7.69
2	Trim 72h	53.85
3	Trim 0h	30.58
3	Trim 120h	13.22
3	Trim 24h	27.27
3	Trim 72h	28.93
4	Trim 0h	33.33
4	Trim 120h	16.33
4	Trim 24h	29.25
4	Trim 72h	21.09

2. Circlized Dendrograms Results

Figures of Circlized dendrogram results for Carbohydrates and Fatty Acids to 0 h, 24 h, 72 h, 120 h to Azithromycin, Oxacillin, and Trimethoprim/ Sulfamethoxazole.

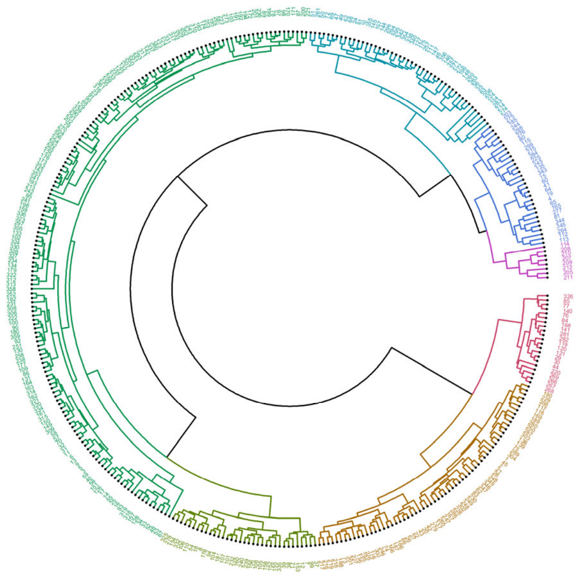

Figure S7. Circlized dendrogram results for carbohydrates region into the FTIR spectra for all samples of *S. aureus* treated with Azithromycin (Azy) at 24 h, 72 h, 120 h and the average MRSA samples with no antibiotic exposure (0 h).

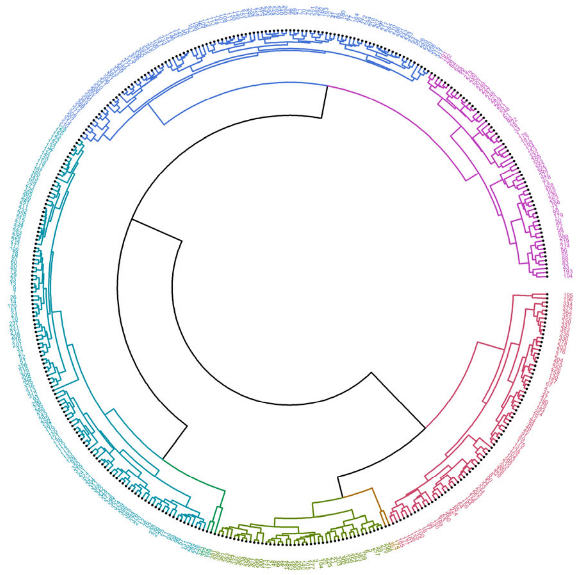

Figure S8. Circlized dendrogram results for carbohydrates region into the FTIR spectra for all samples of *S. aureus* treated with Oxacillin (Oxa) at 24 h, 72 h, 120 h and the average MRSA samples with no antibiotic exposure (0 h).

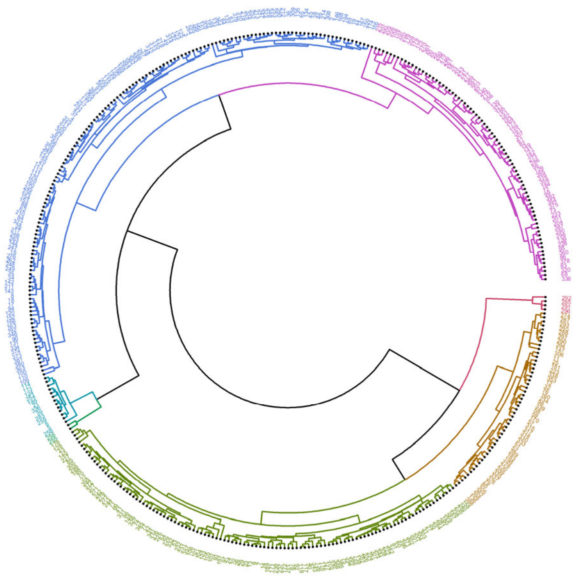

Figure S9. Circlized dendrogram results for carbohydrates region into the FTIR spectra for all samples of *S. aureus* treated with Trimethoprim/Sulfamethoxazole (Trim) at 24 h, 72 h, 120 h and the average MRSA samples with no antibiotic exposure (0 h).

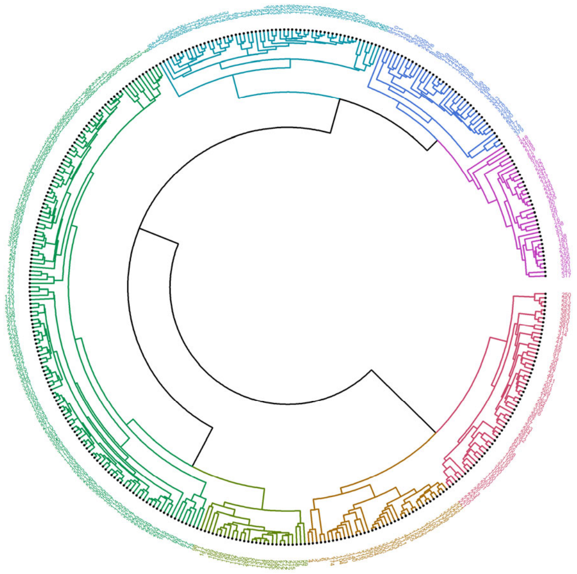

Figure S10. Circlized dendrogram results for fatty acids region into the FTIR spectra for all samples of *S. aureus* treated with Azithromycin (Azy) at 24 h, 72 h, 120 h and the average MRSA samples with no antibiotic exposure (0 h).

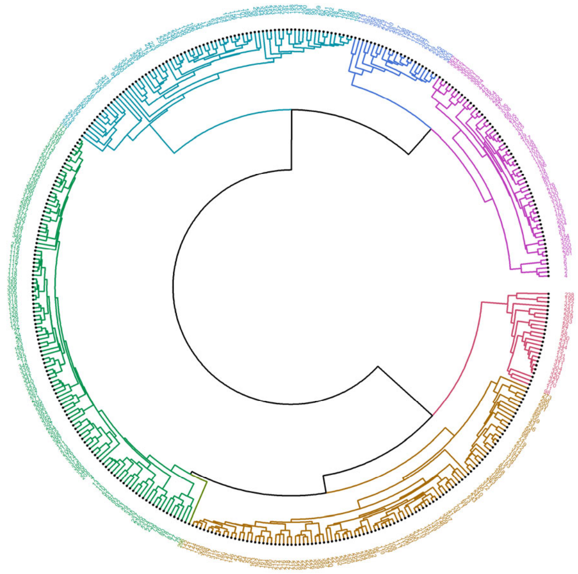

Figure S11. Circlized dendrogram results for fatty acids region into the FTIR spectra for all samples of *S. aureus* treated with Oxacillin (Oxa) at 24 h, 72 h, 120 h and the average MRSA samples with no antibiotic exposure (0 h).

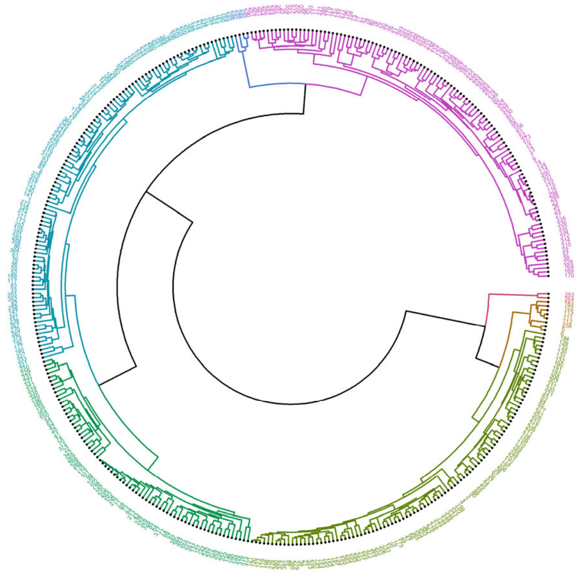

Figure S12. Circlized dendrogram results for fatty acids region into the FTIR spectra for all samples of *S. aureus* treated with Trimethoprim/Sulfamethoxazole (Trim) at 24 h, 72 h, 120 h and the average MRSA samples with no antibiotic exposure (0 h).

PCA

Figures of results of PCA-center and PCA results for Carbohydrates and Fatty acid to 0 h, 24 h, 72 h, 120 h to Azithromycin, Oxacillin, and Trimethoprim/ Sulfamethoxazole, and for all non-methicillin-resistant *S. aureus* samples (iB). Each data group analyzed into

carbohydrates and fatty acids regions at 0 h, 24 h, 72 h, 120 h and iB contains one hundred FTIR spectra samples. For more details about calculation please see methodology section into the main body article.

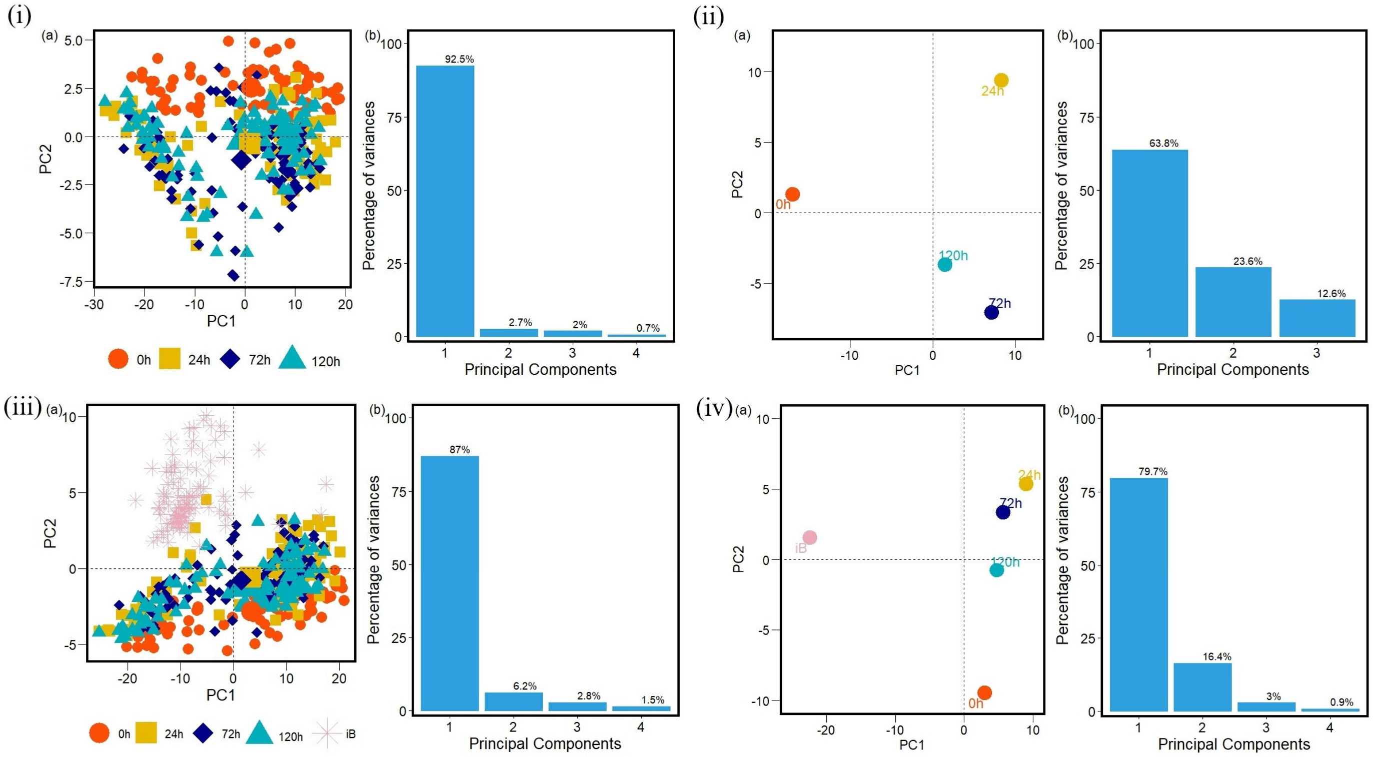

Figure S13. (i) Principal component analysis PCA (a) with variances in (b) and (ii) PCA-center calculations results to carbohydrates region into the FTIR spectra for MRSA samples with no antibiotic exposure (0 h), and for all samples of MRSA treated with Azithromycin (Azy) at 24h, 72h, 120h. (iii,iv) PCA and PCA-center calculation results obtained to carbohydrates region into the FTIR spectra for MRSA samples with no antibiotic exposure (0 h), for all samples of MRSA treated with Azithromycin (Azy) at 24 h, 72 h, 120 h, and for all non-methicillin-resistant *S. aureus* samples (iB). Each data group analyzed into carbohydrates region at 0 h, 24 h, 72 h, 120 h and iB contains one hundred FTIR spectra samples.

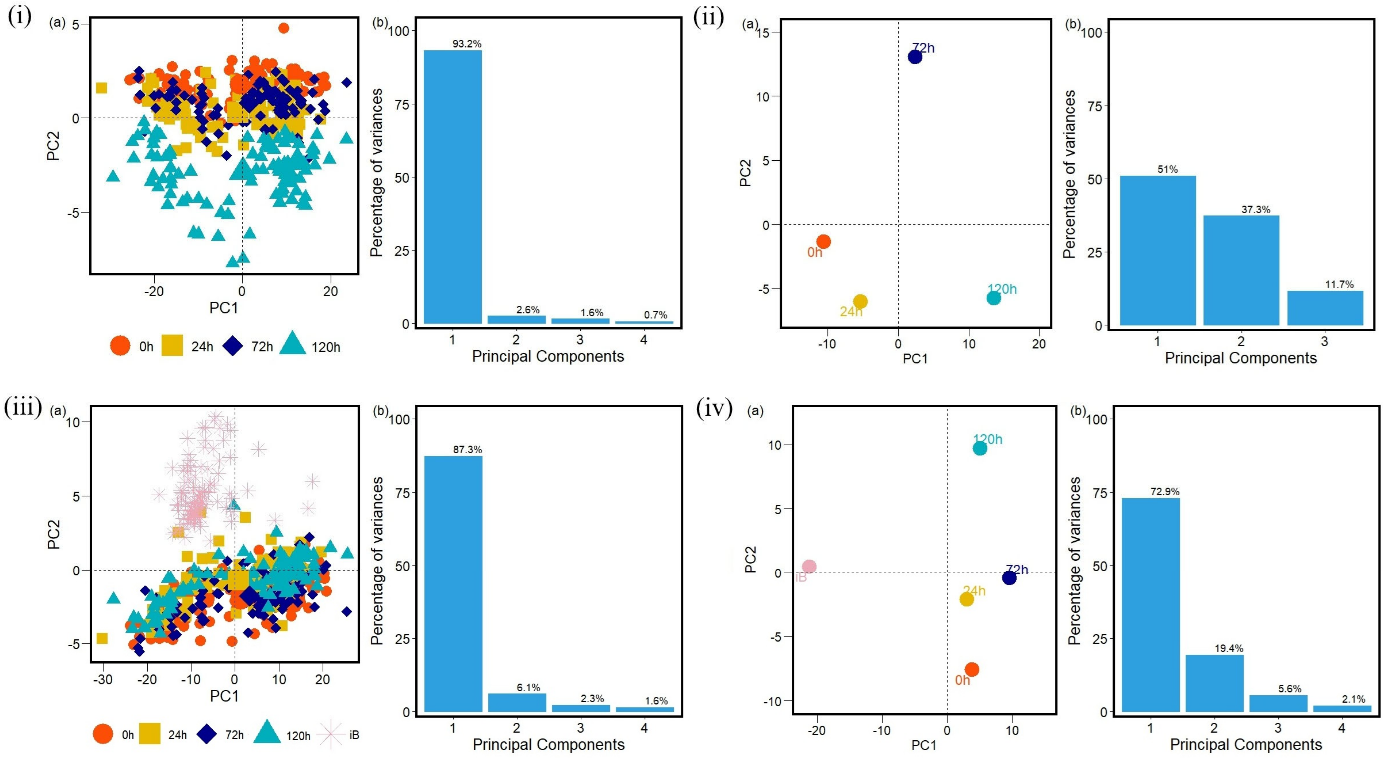

Figure S14. (i) Principal component analysis PCA (a) with variances in (b) and (ii) PCA-center calculations results to carbohydrates region into the FTIR spectra for MRSA samples with no antibiotic exposure (0 h), and for all samples of MRSA treated with Oxacillin (Oxa) at 24 h, 72 h, 120 h. (iii,iv) PCA and PCA-center calculation results obtained to carbohydrates region into the FTIR spectra for MRSA samples with no antibiotic exposure (0 h), for all samples of MRSA treated with Oxacillin (Oxa) at 24 h, 72 h, 120 h, and for all non-methicillin-resistant *S. aureus* samples (iB). Each data group analyzed into carbohydrates region at 0 h, 24 h, 72 h, 120 h and iB contains one hundred FTIR spectra samples.

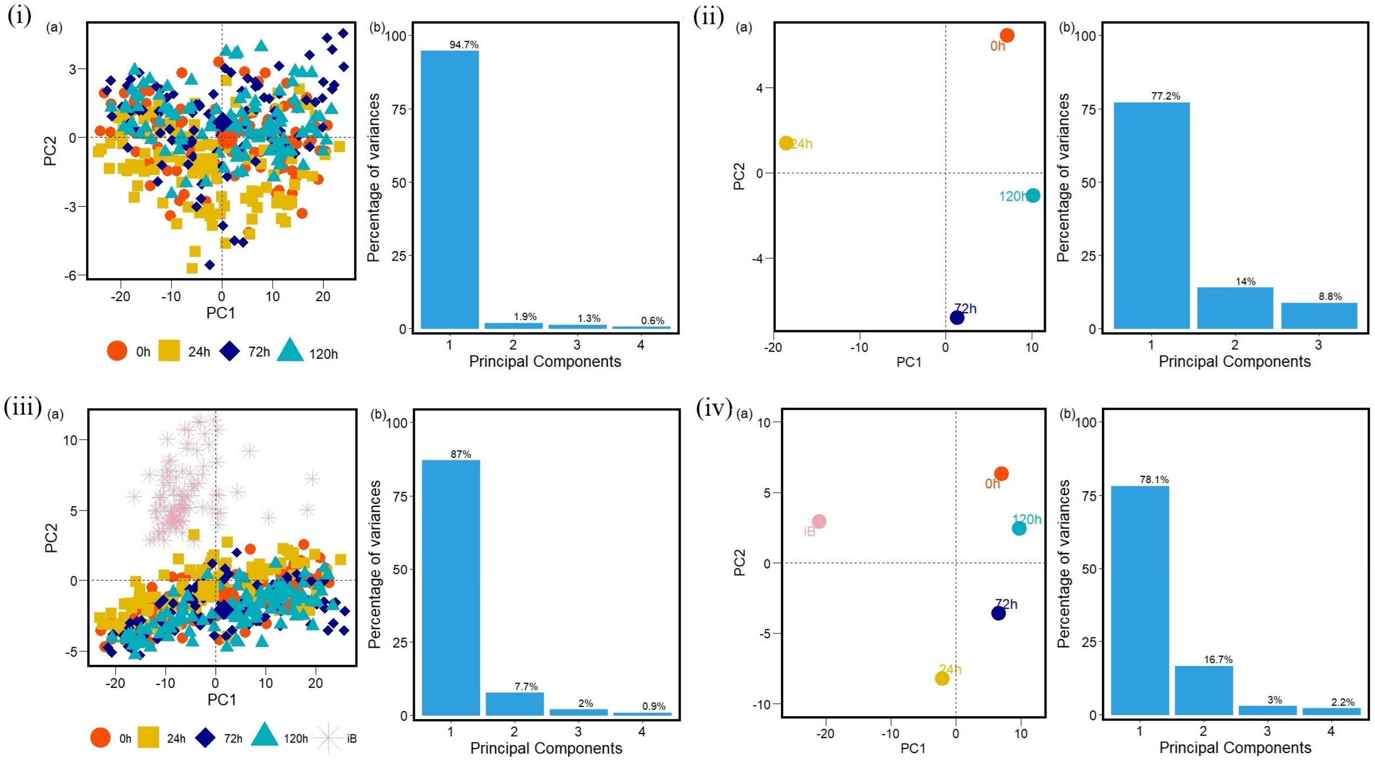

Figure S15. (i) Principal component analysis PCA (a) with variances in (b) and (ii) PCA-center calculations results to carbohydrates region into the FTIR spectra for MRSA samples with no antibiotic exposure (0 h), and for all samples of MRSA treated with Trimethoprim/ Sulfamethoxazole (Trim) at 24 h, 72 h, 120 h. (iii,iv) PCA and PCA-center calculation results obtained to carbohydrates region into the FTIR spectra for MRSA samples with no antibiotic exposure (0 h), for all samples of MRSA treated Trimethoprim/ Sulfamethoxazole (Trim) at 24 h, 72 h, 120 h, and for all non-methicillin-resistant *S. aureus* samples (iB). Each data group analyzed into carbohydrates region at 0 h, 24 h, 72 h, 120 h and iB contains one hundred FTIR spectra samples.

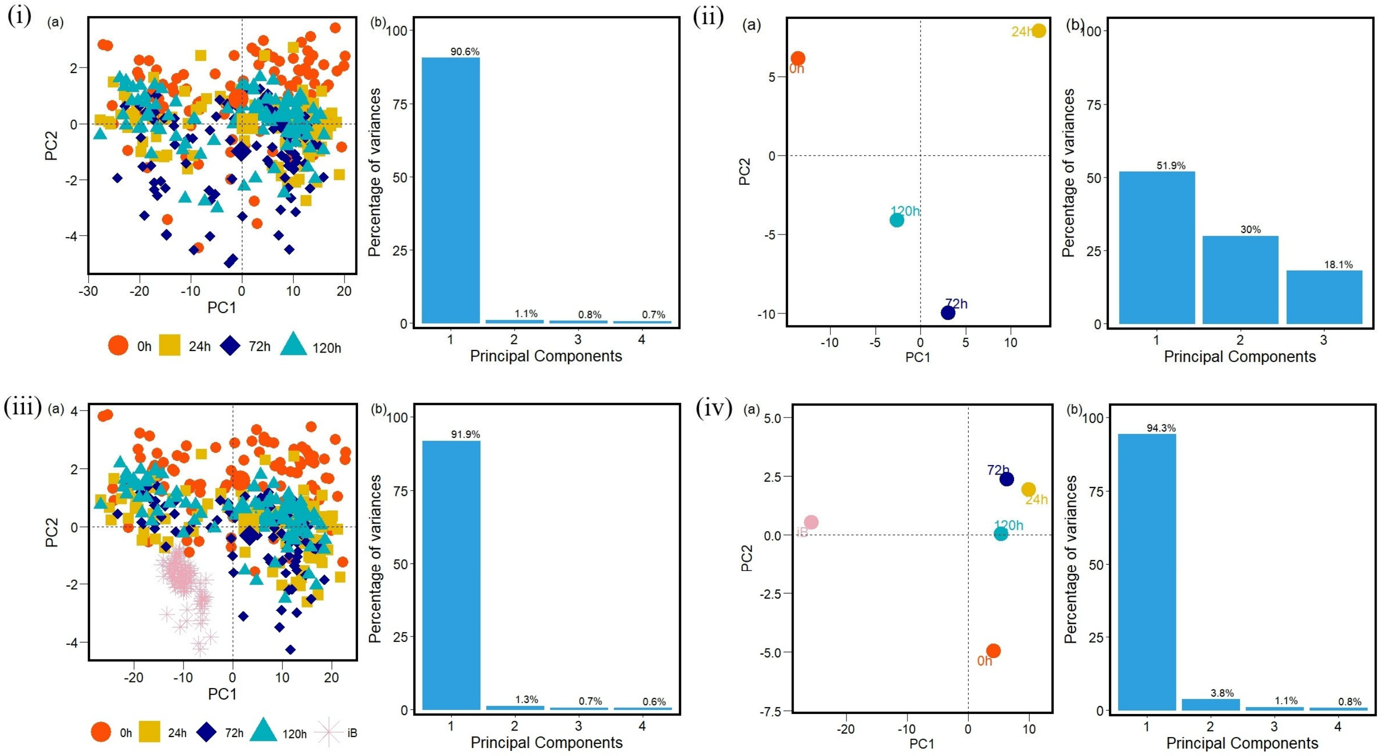

Figure S16. (i) Principal component analysis PCA (a) with variances in (b) and (ii) PCA-center calculations results to fatty acids region into the FTIR spectra for MRSA samples with no antibiotic exposure (0 h), and for all samples of MRSA treated with Azithromycin (Azy) at 24 h, 72 h, 120 h. (iii,iv) PCA and PCA-center calculation results obtained to fatty acids region into the FTIR spectra for MRSA samples with no antibiotic exposure (0 h), for all samples of MRSA treated with Azithromycin (Azy) at 24 h, 72 h, 120 h, and for all non-methicillin-resistant *S. aureus* samples (iB). Each data group analyzed into fatty acids region at 0 h, 24 h, 72 h, 120 h and iB contains one hundred FTIR spectra samples.

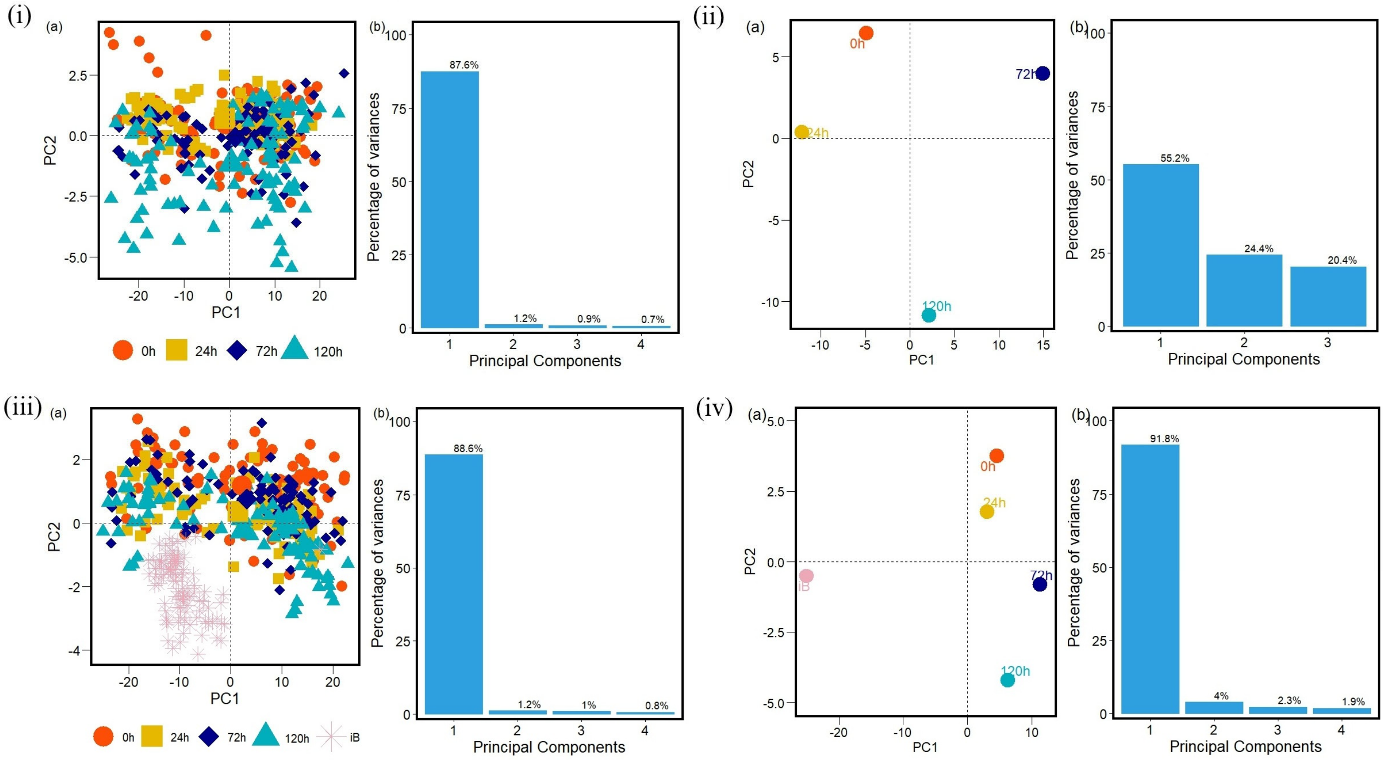

Figure S17. (i) Principal component analysis PCA (a) with variances in (b) and (ii) PCA-center calculations results to fatty acids region into the FTIR spectra for MRSA samples with no antibiotic exposure (0 h), and for all samples of MRSA treated with Oxacillin (Oxa) at 24 h, 72 h, 120 h. (iii,iv) PCA and PCA-center calculation results obtained to fatty acids region into the FTIR spectra for MRSA samples with no antibiotic exposure (0 h), for all samples of MRSA treated with Oxacillin (Oxa) at 24 h, 72 h, 120 h, and for all non-methicillin-resistant *S. aureus* samples (iB). Each data group analyzed into fatty acids region at 0 h, 24 h, 72 h, 120 h and iB contains one hundred FTIR spectra samples.

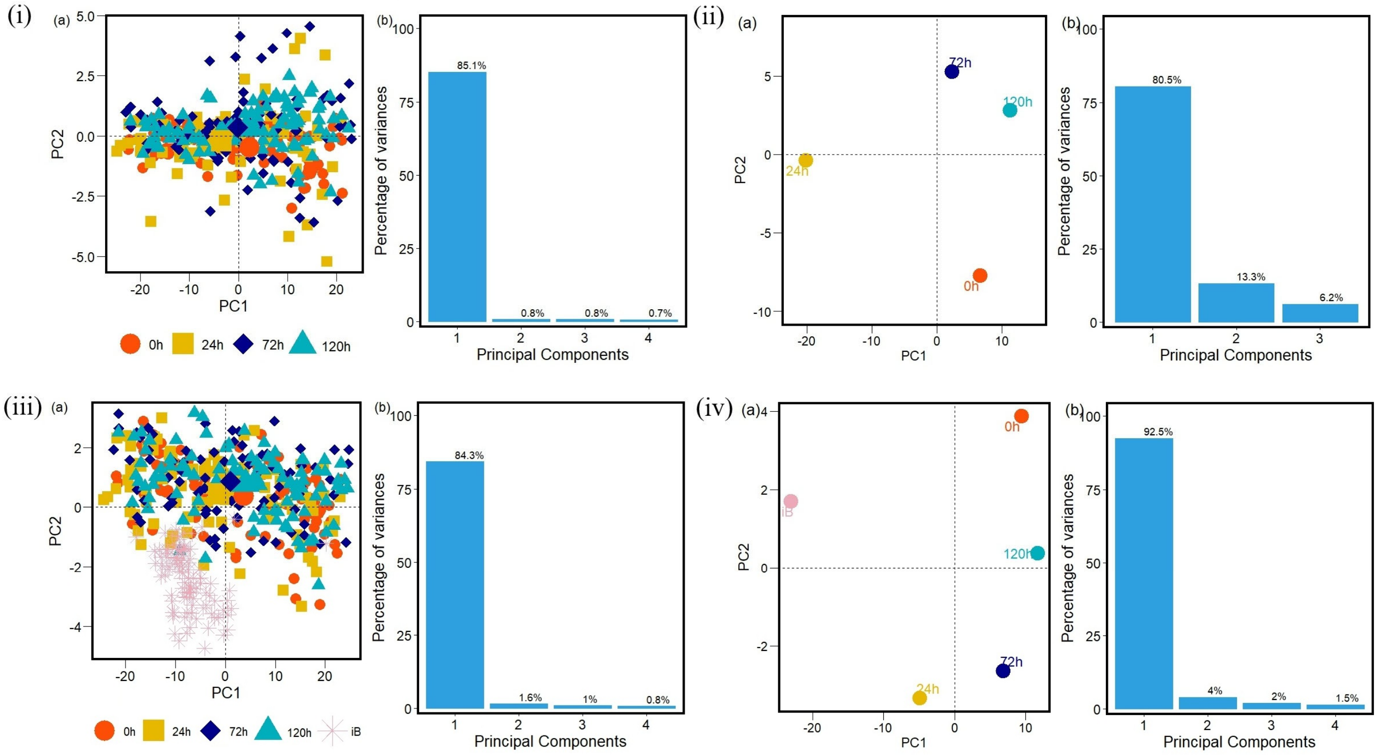

Figure S18. (i) Principal component analysis PCA (a) with variances in (b) and (ii) PCA-center calculations results to fatty acids region into the FTIR spectra for MRSA samples with no antibiotic exposure (0 h), and for all samples of MRSA treated with Trimethoprim/ Sulfamethoxazole (Trim) at 24 h, 72 h, 120 h. (iii,iv) PCA and PCA-center calculation results obtained to fatty acids region into the FTIR spectra for MRSA samples with no antibiotic exposure (0 h), for all samples of MRSA treated Trimethoprim/ Sulfamethoxazole (Trim) at 24 h, 72 h, 120 h, and for all non-methicillin-resistant *S. aureus* samples (iB). Each data group analyzed into fatty acids region at 0 h, 24 h, 72 h, 120 h and iB contains one hundred FTIR spectra samples.
